# BIN2 negatively regulates plant defence against *Verticillium dahliae in Arabidopsis* and cotton

**DOI:** 10.1111/pbi.13640

**Published:** 2021-06-11

**Authors:** Yun Song, Yaohua Zhai, Linxuan Li, Zhaoen Yang, Xiaoyang Ge, Zuoren Yang, Chaojun Zhang, Fuguang Li, Maozhi Ren

**Affiliations:** ^1^ Zhengzhou Research Base State Key Laboratory of Cotton Biology Zhengzhou University Zhengzhou China; ^2^ Institute of Cotton Research Chinese Academy of Agricultural Sciences Anyang China; ^3^ School of Life Sciences Liaocheng University Liaocheng China; ^4^ Institute of Urban Agriculture Chinese Academy of Agricultural Sciences Chengdu China

**Keywords:** BIN2, JAZ, *Verticillium dahlia*, *Arabidopsis*, cotton

## Abstract

*Verticillium* wilt is caused by the soil‐borne vascular pathogen *Verticillium dahliae*, and affects a wide range of economically important crops, including upland cotton (*Gossypium hirsutum*). Previous studies showed that expression levels of *BIN2* were significantly down‐regulated during infestation with *V. dahliae*. However, the underlying molecular mechanism of BIN2 in plant regulation against *V. dahliae* remains enigmatic. Here, we characterized a protein kinase *GhBIN2* from *Gossypium hirsutum*, and identified *GhBIN2* as a negative regulator of resistance to *V. dahliae*. The *Verticillium* wilt resistance of *Arabidopsis* and cotton were significantly enhanced when *BIN2* was knocked down. Constitutive expression of *BIN2* attenuated plant resistance to *V. dahliae*. We found that BIN2 regulated plant endogenous JA content and influenced the expression of JA‐responsive marker genes. Further analysis revealed that BIN2 interacted with and phosphorylated JAZ family proteins, key repressors of the JA signalling pathway in both *Arabidopsis* and cotton. Spectrometric analysis and site‐directed mutagenesis showed that BIN2 phosphorylated AtJAZ1 at T196, resulting in the degradation of JAZ proteins. Collectively, these results show that BIN2 interacts with JAZ proteins and plays a negative role in plant resistance to *V. dahliae*. Thus, BIN2 may be a potential target gene for genetic engineering against *Verticillium* wilt in crops.

## Introduction

*Verticillium* wilt, a highly destructive vascular plant disease, is primarily caused by the soil‐borne plant pathogenic fungus *Verticillium dahliae* (Deketelaere *et al*., 2017; Fradin and Thomma, 2006). This vascular pathogen can attack a wide spectrum of globally important crops including cotton (*Gossypium* spp.) and cause tremendous economic losses (Pegg *et al*., 2002; Yang *et al*., 2015). Because of the long‐term viability *V. dahliae* in soil, the lack of resistance genes in upland cotton (*G. hirsutum*), *Verticillium* wilt is notoriously difficult to prevent and control, earning it the name ‘cancer of cotton’ (Aguado *et al*., 2008; Su *et al*., 2018; Zhang *et al*., 2012). To respond to fungal and other pathogens, plants have evolved a wide spectrum of defences, including physical and chemical barriers, and some inducible responses (López *et al*., 2008; Pieterse *et al*., 2009). Among these, phytohormones play a crucial role in protecting plants from fungal pathogen infestation.

Phytohormones brassinosteroids (BRs) function as master regulators of plant immunity (Anwar *et al*., 2018; Nawaz *et al*., 2017; Wang, 2012; Yu *et al*., 2018). BRs were perceived by the receptor‐like kinase BRASSINOSTEROID INSENSITIVE1 (BRI1) and activated a signal transduction cascade to regulate BR‐responsive genes (Belkhadir and Jaillais, 2015; Kim and Wang, 2010). Exogenous application of low concentrations of BR significantly improves the resilience of cucumber (*Cucumis sativus* L.), tobacco (*Nicotiana tabacum* L.), tomato (*Solanum lycopersicum*), and cotton (*Gossypium* spp.) to viral and fungal pathogens (Bajguz and Hayat, 2009; Gao *et al*., 2013; Wang *et al*., 2015). Several studies reported that BRs modulated plant immunity by regulating the JA signalling pathway. For instance, the BR‐mediated susceptibility to Rice Black‐Streaked Dwarf Virus (RBSDV) infection can be suppressed by the JA‐mediated defence in rice (*Oryza sativa*) (He *et al*., 2017). *Nicotiana attenuated* BAK1, a co‐receptor of BR receptor BRI1, is involved in modulating herbivory‐induced JA accumulation (Yang *et al*., 2011). The GSK3‐like kinase BIN2 (Brassinosteroid insensitive 2) is a key negative regulator of the BR signalling pathway. BIN2 phosphorylates and destabilizes the two homologous transcription factors BRASSINAZOLE RESISTANT1 (BZR1) and BZR2/BES1 to block BR signalling pathway (Li and Nam, 2002). The GSK3‐like kinase BIN2 is involved in plant multiple developmental and physiological processes (Saidi *et al*., 2012; Tong and Chu, 2018; Youn and Kim, 2015). Previous study had implicated that *BIN2* expression was inhibited by *V. dahliae* infestation (Gao *et al*., 2013). However, the molecular mechanism of GSK3‐like kinase BIN2‐mediated antifungal defence remains unclear in plants.

Recent genetic evidence indicates that jasmonic acid (JA) biosynthesis and signalling pathway are integral to plant resistance to *V. dahliae* (Du *et al*., 2017; Goossens *et al*., 2016; Howe *et al*., 2018; Wang *et al*., 2019; Wasternack and Song, 2016). The JA signals are sensed by COI1‐JAZ (CORONATINE INSENSITIVE‐JASMONATE ZIM DOMAIN) co‐receptors. Within the signalling cascade, JAZ proteins are degraded through the 26S proteasome, and then activate JA‐related transcription factors (such as MYC2, MYC3, and MYC4), which subsequently regulate the downstream signalling cascades and modulate the respective plant responses (Chini *et al*., 2007; Sheard *et al*., 2010; Thines *et al*., 2007; Xu *et al*., 2002; Yan *et al*., 2007). Previous studies in *Arabidopsis* showed that unobstructed JA signal transduction helps to improve plant resistance to *V. dahliae* (Fradin *et al*., 2011). Mutants impaired in the JA signalling pathways become susceptible to *V. dahliae* infection (Campos *et al*., 2014; De Geyter *et al*., 2012; Fradin *et al*., 2011; Jiang and Yu, 2016; Thaler *et al*., 2004). During *V. dahliae* attack, JA and JA‐Ile are significantly accumulated in plants, followed by increased expression of JA signal transduction genes (Hu *et al*., 2018a). JA largely contributes to cotton resistance to *V. dahliae* (Gao *et al*., 2013). Reduced transcription levels of *GhSSN* (SILENCE‐INDUCED STEM NECROSIS) induce the accumulation of JA and JA‐Ile and thus enhance cotton immunity to *Verticillium* wilt (Sun *et al*., 2014). GhCPK33 negatively regulates cotton sensitivity to *V. dahliae* by phosphorylating GhOPR3, a pivotal node gene of JA biosynthesis (Hu *et al*., 2018b). In *Arabidopsis*, constitutive JAZ expression increases susceptibility to the fungal pathogen (Thatcher *et al*., 2016). *Gossypium barbadense* WRKY1 transcription factor attenuates cotton resistance to *V. dahliae* by promoting *JAZ1* expression (Li *et al*., 2014). GhJAZ2 protein interacts with GhbHLH171 and inhibits its transcriptional activity, eventually restraining the JA‐mediated defence against *V. dahliae* (He *et al*., 2018).

In this study, we identified the protein kinase BIN2 as a negative regulator of defence against *V. dahliae* in *Arabidopsis* and cotton. We provide evidence that BIN2 interacted with and phosphorylated JAZ proteins, which are negative regulators of the JA signalling pathway. We demonstrate that BIN2 phosphorylation destabilized JAZ1. Our study provides novel insights into the phosphorylation network of the BIN2 protein and the interaction between the BR and JA signalling pathways. In conclusion, this study showed that BIN2 directly participates in the regulation of plant biotic stress response and could be a potential molecular target for engineering next‐generation cotton crops with improved resistance against *Verticillium* wilt disease.

## Results

### Identification of the *GhBIN2* gene and its expression pattern

**Figure 1:**
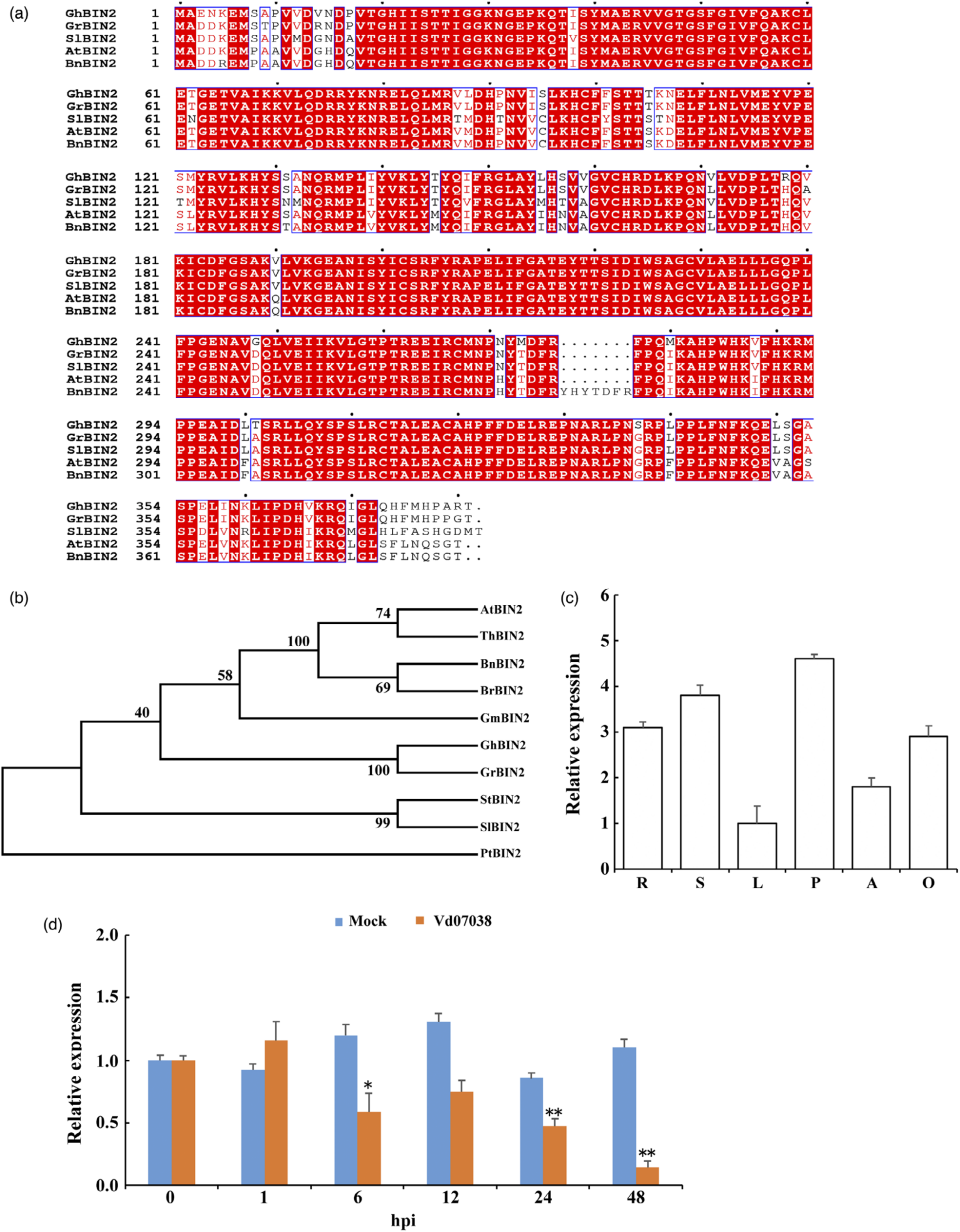
Sequence, phylogenetic, and expression analysis of GhBIN2. (a) Amino acid sequences alignment of GhBIN2 and functionally characterized BIN2s from other representative organisms. The alignment was performed by ClustalW and drawn using ESPript version 3.0. The invariant residues were coloured in red and shadowed, similar residues were in red boxes. (b) Phylogenetic analysis of GhBIN2 and BIN2s from other plants. The neighbour‐joining phylogenetic tree was generated using the MEGA5.0 program. (c) Analysis of *GhBIN2* expression in different tissues measured by QRT‐PCR. Total RNA was isolated from roots (R), stems (S), leaves (L), petals (P), anthers (An), and ovules (O) of *Gossypium hirsutum* L. cv ZM24. The *GhHistone3* gene was used as the internal control gene. (d) Transcript level analysis of *GhBIN2* in cotton roots inoculated with *V. dahliae* measured by QRT‐PCR. The roots of 14‐day‐old seedlings at 0–48 h post‐inoculation (hpi) were harvested for total RNA extraction. Error bars represent ±SD for three independent experiments. BIN2 sequences were obtained for *Gossypium hirsutum* (GhBIN2, GenBank: KM453729), *Arabidopsis* (AtBIN2, TAIR: AT4G18710), *Solanum tuberosum* (StBIN2, GenBank: MH341405), *Pinus taeda* (PtBIN2, GenBank: MH017211), *Glycine max* (GmBIN2, GenBank: MF457588), *Thellungiella halophile* (ThBIN2, GenBank: AK352525), *Solanum lycopersicum* (SlBIN2, GenBank: NM_001356270), *Brassica napus* (BnBIN2, GenBank: DQ767688), *Brassica rapa* (BrBIN2, GenBank: XM_009133885), and *Gossypium raimondii* (GrBIN2, GenBank: XM_012594385).

BIN2 is a negative regulator of the BR signalling pathway (Li and Nam, 2002; Li *et al*., 2001; Xiong *et al*., 2017). To dissect the function of cotton BIN2, we identified a *BIN2* gene (Accession number in GenBank: KM453729) in the upland cotton (*Gossypium hirsutum*) genome. The full‐length cDNA of *GhBIN2* consists of 1143 nucleotides and encodes a protein of 381 amino acids. There were five homologs of *GhBIN2* in the upland cotton genome (Table S1). We performed multiple sequence alignment with GhBIN2 and other BIN2s from various plant species (Figure 1a). GhBIN2 showed high identity scores with BIN2 proteins from other species. The phylogenetic analysis with the protein sequences indicated that GhBIN2 was evolutionarily conserved, and the closest ortholog of *GhBIN2* was GrBIN2 (*Gossypium raimondii*) (Figure 1b). Furthermore, we analysed the expression of *GhBIN2* in various tissues of the cotton plant by QRT‐PCR. Expression of the *GhBIN2* gene was ubiquitous in cotton plants and showed relatively higher levels in petals, stems, and roots compared with those in leaves, anthers, and ovules (Figure 1c). To investigate the involvement of GhBIN2 in disease responses, we examined the expression pattern of *GhBIN2* in cotton roots after inoculation with *V. dahliae* (Figure 1d). *GhBIN2* transcript abundance was down‐regulated in roots after inoculation with *V. dahliae*. These results suggested that GhBIN2 was evolutionarily conserved and may be involved in the cotton defence against *V. dahliae* infection.

### Silencing *GhBIN2* expression increased cotton resistance to *V. dahliae* colonization

**Figure 2:**
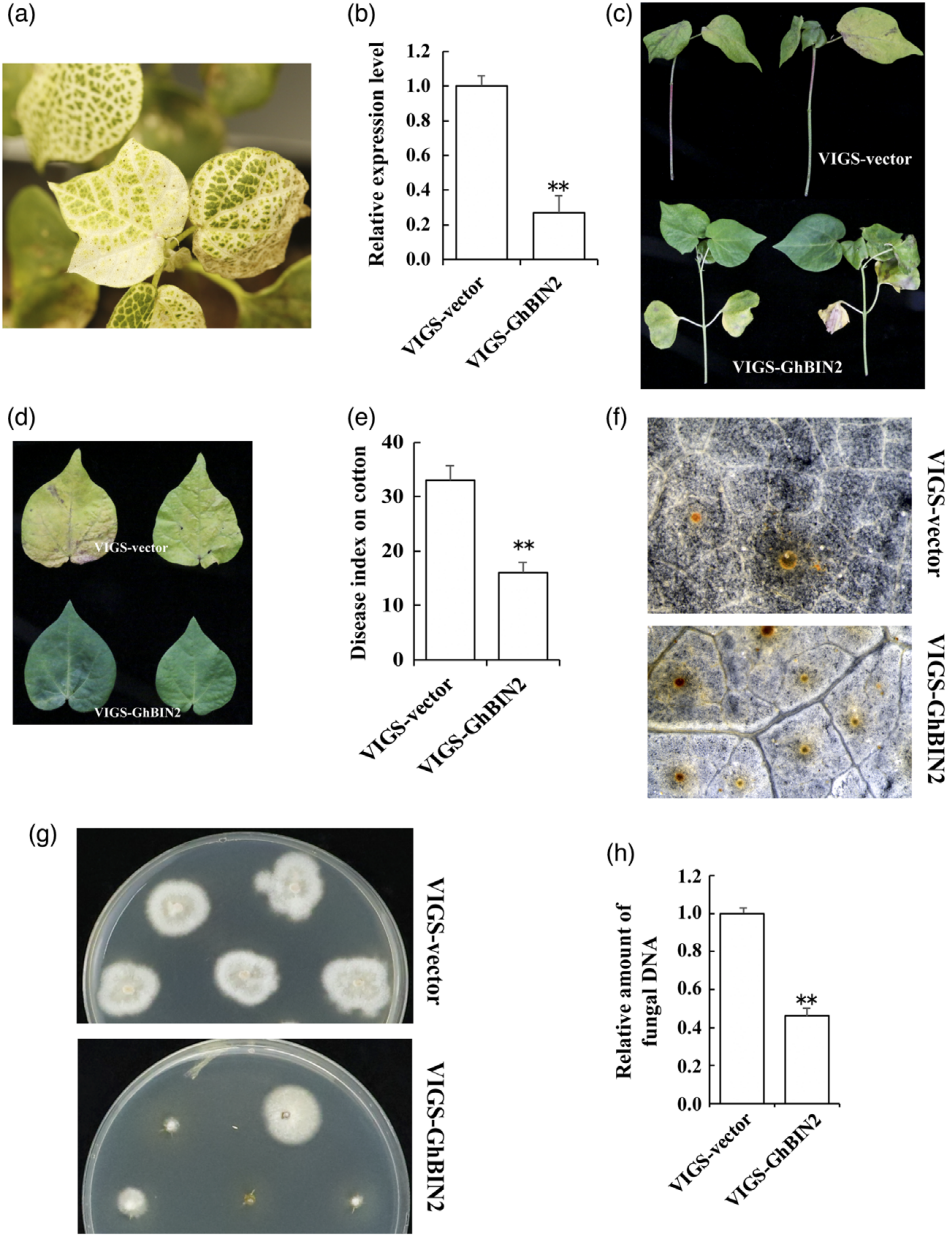
Reduced susceptibility of *GhBIN2*‐silenced cotton plants to *V*. *dahlia*. (a) Photobleaching phenotype of the plants inoculated with TRV:PDS for 16 days. (b) Validation of the efficiency of VIGS vector TRV:*GhBIN2* in injected plants by QRT‐PCR analysis. (c) Disease symptoms of TRV:00‐ and TRV:*GhBIN2*‐injected plants infected by *V. dahliae* strain 07038 at 20 days post‐inoculation (dpi). (d) Phenotypes of TRV:00‐ and TRV:*GhBIN2*‐injected cotton leaves infected by *V. dahliae* at 20 dpi. (e) Disease index of the TRV:00‐ and TRV:*GhBIN2*‐injected plants at 25 dpi. (f) ROS response observed by DAB‐peroxidase staining in leaves of TRV:00‐ and TRV:*GhBIN2*‐injected plants after inoculated with *V*. *dahliae* for 24 h. (g) Stem sections at 20 dpi were plated on potato dextrose agar medium and incubated at 25 °C. Photographs were taken 5 days after plating. (h) The relative amount of fungus determined by QRT‐PCR in TRV:00‐ and TRV:*GhBIN2*‐injected plants at 25 dpi. Error bars represent ±SD (*n* = 3). Each independent experiment contains 10 plants per treatment. Asterisks (*P < 0.05, **P < 0.01) indicate significant differences between TRV:00 and TRV:*GhBIN2*‐injected plants.

In the current study and previous research, *BIN2* was down‐regulated after inoculation with *V. dahliae* (Figure 1d; Gao *et al*., 2013). To better understand the putative function of GhBIN2 during the immune response against *V. dahliae*, we employed the tobacco rattle virus (TRV)‐based virus‐induced gene silencing (VIGS) strategy to knock down the expression of *GhBIN2*. When the leaves of cotton seedlings inoculated with pTRV:*GhPDS* showed a photobleaching phenotype (Figure 2a), the TRV:00 and TRV:*GhBIN2* cotton seedlings were harvested to determine the transcript levels of *GhBIN2* (Figure 2b). The QRT‐PCR results showed that the transcript abundance of *GhBIN2* was significantly reduced in TRV:*GhBIN2* plants, suggesting that *GhBIN2* was successfully silenced in TRV:*GhBIN2* plants. Two weeks after *Agrobacterium* infiltration, these cotton seedlings were challenged with the *V. dahliae* strain Vd07038 and monitored for the development of disease symptoms. We found that knock‐down of *GhBIN2* enhanced plant resistance to *V. dahliae*. Defoliation and yellowing disease symptoms were more severe in the TRV:00 plants than in the TRV:*GhBIN2* plants. (Figure 2c and d). The disease index in the TRV:*GhBIN2* plants was much lower than that of the control seedlings (Figure 2e). Reactive oxygen species (ROS) burst is one of the canonical defence events during plant immune response (Hancock *et al*., 2002). We checked the ROS levels in the infected cotton leaves by DAB‐peroxidase staining. The TRV:*GhBIN2* cotton seedlings accumulated more spots of brown precipitates than did the TRV:00 plants, indicating a stronger defence response through the ROS pathway (Figure 2f). We performed a recovery assay from stem sections of the inoculated plants to examine the extent of *V. dahliae* colonization. Significantly fewer fungal colonies were present on the TRV:*GhBIN2* plants than on the control plants (Figure 2g). Fungal DNA can be measured using QRT‐PCR methods (Atallah *et al*., 2007). We measured the levels of *V. dahliae* in the TRV:*GhBIN2* and TRV:00 plants. Levels of *V. dahliae* colonization in the TRV: *GhBIN2* plants were lower than in the TRV:00 plants (Figure 2h). These results show that knock‐down of the *GhBIN2* gene enhances the resistance of cotton plants to *V. dahliae* infection.

### *GhBIN2* overexpression attenuates cotton resistance to *Verticillium dahliae*

**Figure 3:**
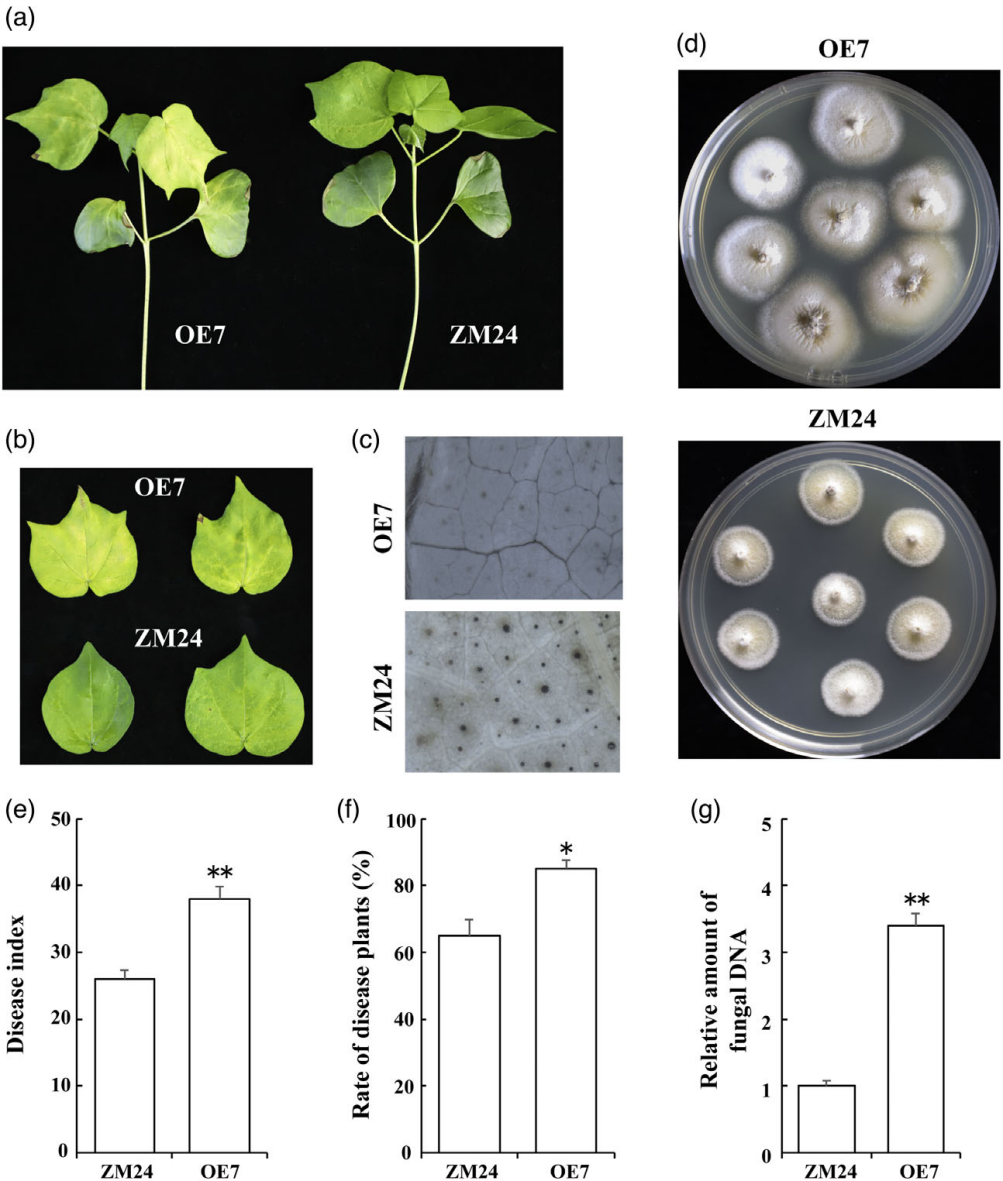
Increased susceptibility to *V. dahliae* in cotton plants overexpressing *GhBIN2*. (a) Symptoms of WT ZM24 and *GhBIN2* overexpression transgenic plants (OE7) inoculated with *V. dahliae* for 20 days. (b) Phenotypes of leaves of WT ZM24 and *GhBIN2*‐overexpressing (OE7) cotton plants inoculated with *V. dahliae* (20 dpi). (c) Accumulation of hydrogen peroxide observed by DAB‐peroxidase staining in WT ZM24 and *GhBIN2*‐overexpressing (OE7) cotton plants after inoculated with *V*. *dahliae* for 24 h. (d) Fungal recovery assay. Stem sections placed on potato dextrose agar medium at 20 dpi. Photographs were taken 5 days after culture. (e) Disease index of the WT ZM24 and *GhBIN2* overexpression transgenic plants (OE7) at 25 dpi. (f) Rate of disease plants in the WT ZM24 and *GhBIN2* overexpression transgenic plants (OE7) at 25 dpi. (g) Relative quantification of *V. dahliae* biomass of the cotton leaves by QRT‐PCR at 25 dpi. Error bars represent ±SD (*n* = 3). Each independent experiment contains 10 plants per treatment. Asterisks (*P < 0.05; **P < 0.01) indicate significant differences from control.

Thirteen independent transgenic cotton lines constitutively overexpressing *GhBIN2* were obtained. Three lines with different expression levels of *GhBIN2* were selected for the following analysis (Figure S1). These *GhBIN2* overexpression (OE) cotton seedlings along with the corresponding WT plants were subjected to challenge with *V. dahliae* (Figure 3). Disease symptoms were examined at 20 days post‐inoculation. *GhBIN2*‐overexpressing cotton plants OE7 displayed more severe wilting and yellowing symptoms than did the control plants (Figures 3a and b). By DAB‐peroxidase staining of WT and OE7 leaves, we observed larger and more dark spots accumulated in WT plants than in OE7 plants (Figure 3c), suggesting that the ROS burst in transgenic plants was inhibited by the overexpression of *GhBIN2*. The fungal recovery assay from the stem sections of inoculated plants showed a greater degree of *V. dahliae* colonization in OE7 (Figure 3d). The disease index (Figure 3e), rate of diseased plants (Figure 3f), and the relative amount of fungal DNA (Figure 3g) were also analysed to investigate the defence behaviour of *GhBIN2*‐transgenic plants. These results showed that *GhBIN2* overexpression in cotton plants resulted in severely compromised resistance to *V. dahliae*.

### BIN2 functions as a negative regulator of plant resistance against *V. dahliae* in *Arabidopsis*

**Figure 4:**
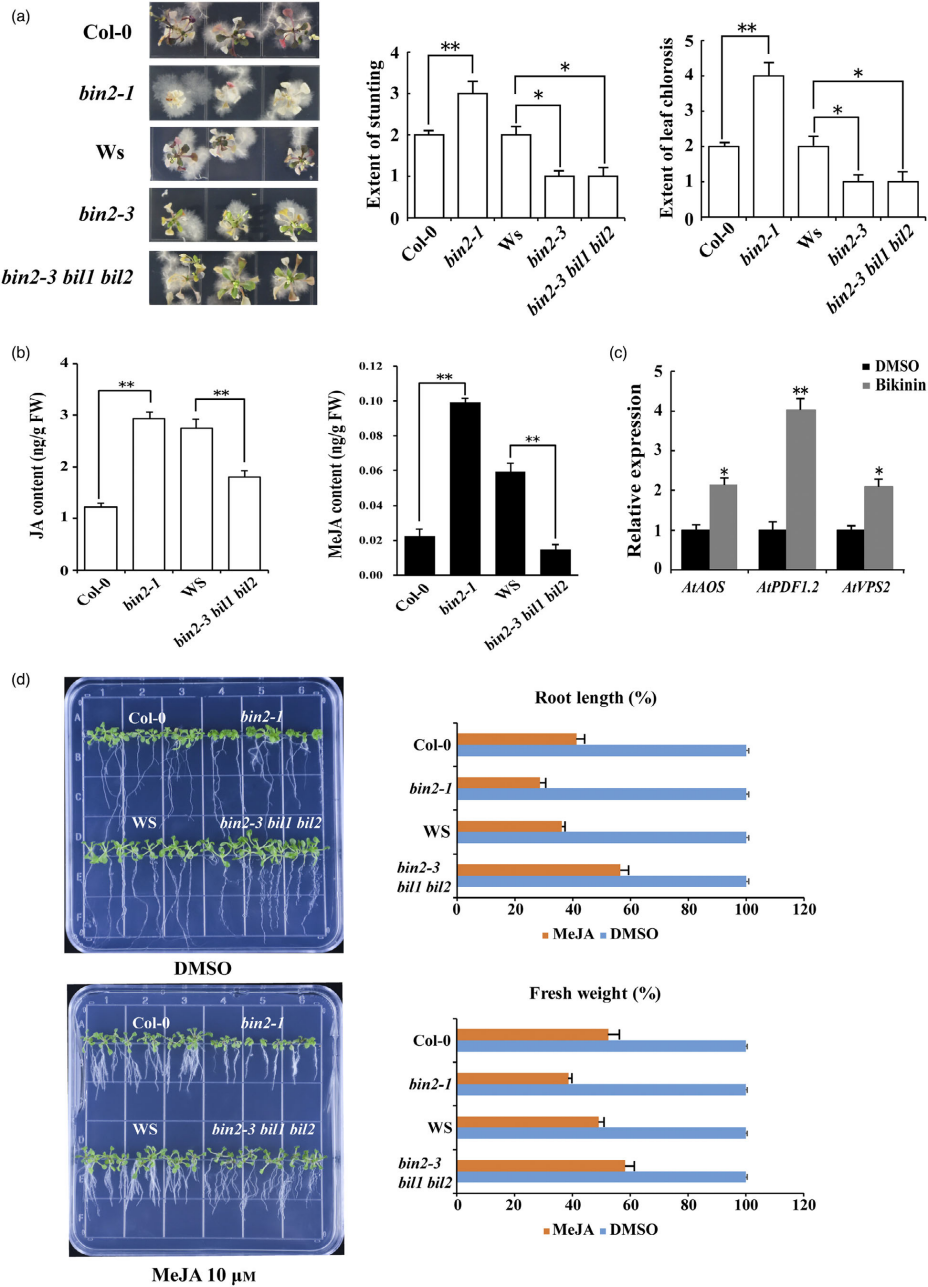
BIN2 is a negative regulator of resistance to *V. dahliae* in *Arabidopsis*. (a) Disease symptoms of WT and AtBIN2‐related mutants inoculated with *V. dahliae*. The *bin2‐1* mutant is the gain‐of‐function mutant of BIN2. The *bin2‐3* and *bin2‐3 bil1 bil2* mutants are the BIN2 and its close homologs BIL1 and BIL2 loss‐of‐function mutants. Two‐week‐old *Arabidopsis* plants were inoculated with *V. dahliae*, and photographs were taken at 14 dpi. The extent of stunting and leaf chlorosis in these *Arabidopsis* plants at 14 dpi were investigated. (b) Measurement of JA and MeJA contents in two‐week‐old WT and AtBIN2‐related mutants. Data shown are the means from at least 10 seedlings for each indicated plant line. (c) Expression patterns of JA‐related marker genes under BIN2 inhibition. Relative expression levels of two JA responsive genes *AtPDF1.2*, *AtVPS2*, and one JA biosynthetic gene *AtAOS* were examined. The expression levels were assessed by QRT‐PCR using *AtActin2* as the internal control gene. Bikinin was dissolved in DMSO, so the expression levels of these genes in DMSO were set to 1. (d) Phenotypes of 10‐day‐old WT and AtBIN2‐related mutant seedlings exposed to DMSO and 10 μm MeJA. The fresh weight and root length of these seedlings (at least 10 seedlings for each indicated plant line) were measured. Error bars represent ±SD (*n* = 3). Asterisks (*P < 0.05, **P < 0.01) indicate significant differences from the control.

To investigate whether BIN2 performs a similar function across plant species, we evaluated the sensitivity of BIN2 mutants to *V. dahliae* infection in the model plant *Arabidopsis* (Figure 4a). The disease symptoms caused by *V. dahliae* were more severe in the gain‐of‐function mutant *bin2‐1* seedlings compared with the control plants *Col‐0*. The loss‐of‐function mutant *bin2‐3* and *bin2‐3 bil1 bil2* showed enhanced resistance to *V. dahliae* compared with the control plants *Ws* (Jonak and Hirt, 2002). We counted the numbers of stunted and chlorotic leaves in *AtBIN2* mutants and WT plants, and found a relatively lower level of stunting and chlorosis in *AtBIN2* loss‐of‐function mutants and *Col‐0* plants (Figure 4a). Additionally, disease symptoms induced by *V. dahliae* on the rosette leaves of these seedlings grown in the pots showed the same pattern with those grown in the MS medium (Figure S2). These results indicated that BIN2 negatively regulated plant resistance against *V*. *dahliae* in the model plant *Arabidopsis*, which is consistent with the functions of GhBIN2, indicating conserved functions of BIN2 against *Verticillium* wilt from *Arabidopsis* to cotton.

JA plays a vital role in plant response to *V*. *dahliae* (Fradin *et al*., 2011; Goossens *et al*., 2016; Howe *et al*., 2018; Veronese *et al*., 2006; Wasternack and Song, 2016). Importantly, studies have also reported that BRs participate in modulating plant immunity through regulating the JA signalling pathway (He *et al*., 2017; Yang *et al*., 2011). We investigated whether the BR negative regulator BIN2‐mediated resistance to *V*. *dahliae* is related to the JA signalling pathway. First, we examined the endogenous JA and MeJA contents in *AtBIN2* mutants (Figure 4b). We found that BIN2 gain‐of‐function plants caused significant JA and MeJA accumulation, and the production of JA and MeJA was suppressed in the BIN2 loss‐of‐function mutants. Bikinin is one of the most specific inhibitors of BIN2 kinase protein, and previous studies showed that the expression levels of BIN2 or other BR‐related genes were significantly altered by treatment with bikinin (De Rybel *et al*., 2009; Rozhon *et al*., 2019). In the present study, bikinin was used to treat WT seedlings (Figure 4c). More transcripts of JA‐responsive marker genes including *AOS*, *PDF1.2*, and *VPS2* accumulated under BIN2 inhibition. Furthermore, we examined the response of *AtBIN2* mutants to the MeJA treatment (Figure 4d). As indicated by the decreases in root length and fresh weight, *bin2‐1* showed enhanced sensitivity to JA signalling, and the sensitivity of *bin2‐3* and *bin2‐3 bil1 bil2* to JA was reduced. These results suggest that BIN2 negatively regulates plant resistance to *V. dahliae* and affects the JA signalling pathway.

### AtBIN2 physically interacts with AtJAZ proteins

**Figure 5:**
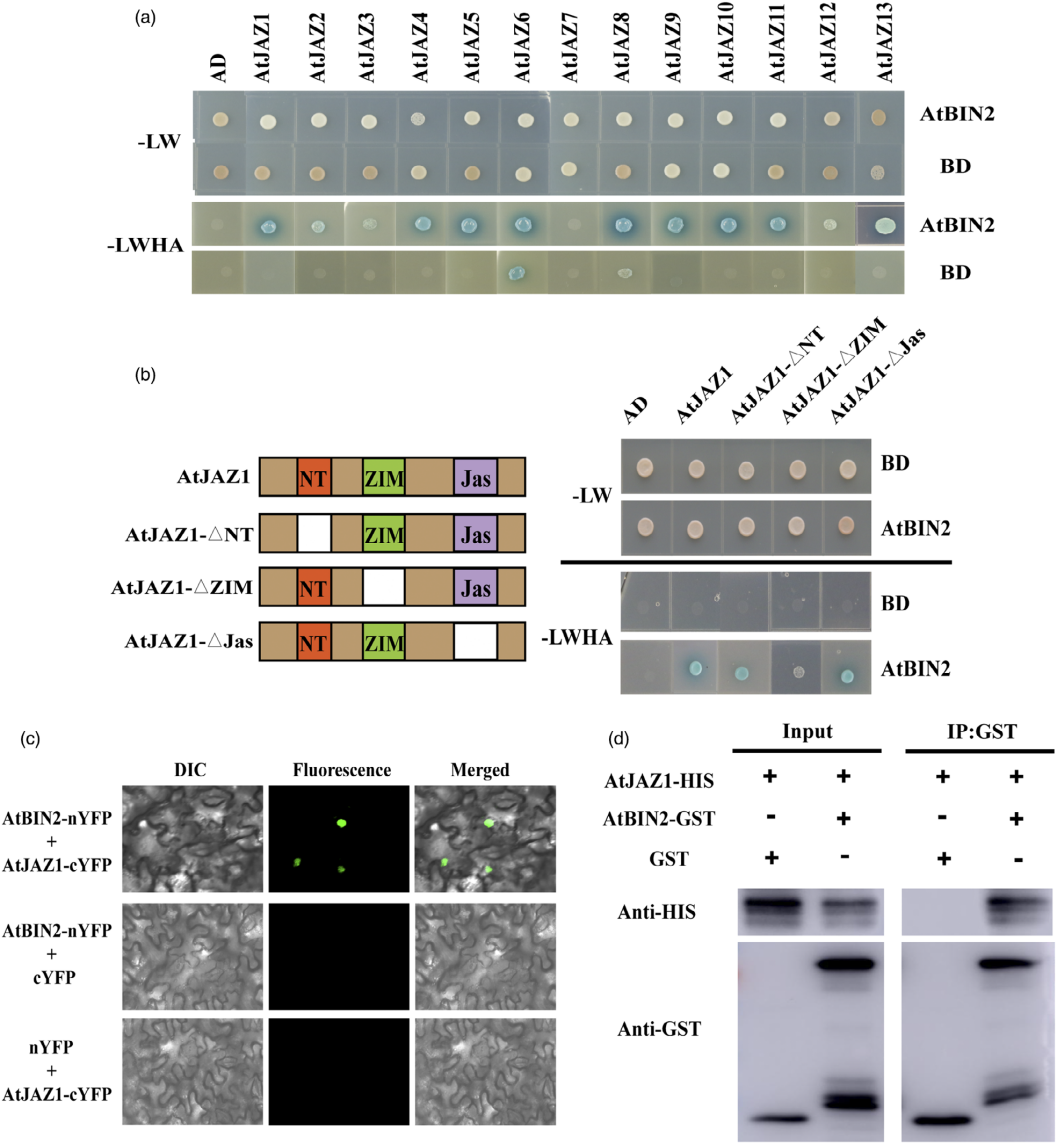
Physical interactions between AtBIN2 with AtJAZ proteins. (a) Two‐hybrid screening between AtBIN2 and AtJAZ proteins. The empty pGADT7 (AD) and pGBKT7 (BD) were used as negative controls. –LW represents SD‐Leu‐Trp plates. –LWHA represents SD‐Leu‐Trp‐His‐Ade plates. (b) Identification of domains required for BIN2‐JAZ1 interaction using the two‐hybrid screening. The ZIM domain of JAZ1 is required for this interaction. Left: schematic representations of JAZ1 and their deletion constructs. Right: results of the two‐hybrid screening. NT, N‐terminal domain; ZIM, zinc‐finger expressed in inflorescence meristem domain; Jas, jasmonate‐associated domain. (c) Bimolecular fluorescence complementation (BiFC) analysis showing the interaction between AtBIN2 and AtJAZ1. The N‐terminal part of YFP was fused with AtBIN2 (AtBIN2‐nYFP), and the C‐terminal part of YFP was fused with AtJAZ1 (AtJAZ1‐cYFP). AtBIN2‐nYFP and AtJAZ1‐cYFP were co‐expressed in *N. benthamiana*. Fluorescence was monitored by confocal microscopy at 48 h post infiltration. (d) Interaction of BIN2 and JAZ1 proteins in a pull‐down assay. The His‐tag JAZ1 protein was incubated with GST‐agarose bound with GST‐BIN2 or GST proteins and was assayed by immunoblotting with anti‐His antibodies.

To understand the molecular mechanism modulated by AtBIN2 in regulating the JA signalling pathway, we used two‐hybrid screening to discover potential BIN2‐interacting proteins (Figure S3A). The *BIN2* coding sequences were constructed into the bait vector pGBKT7. *AtOPR3* and *AtJAR1* genes play important roles in JA biosynthesis, and *AtCOI1*, *AtNINJA*, *AtMYC2*, and *AtJAZ1* are key genes of JA signal transduction (Wasternack and Hause, 2013; Wasternack and Song, 2016). These JA‐related genes were constructed into the vector pGADT7. The JAZ1 protein was identified as an interacting protein of BIN2 in two‐hybrid assays (Figure S3A). Next, we examined the interaction between BIN2 and all JAZ family members by two‐hybrid assays. Except for JAZ7, 12 of the 13 JAZ proteins interacted with BIN2 (Figure 5a). GhBIN2 also interacted with GhJAZ2 protein (Figure S3B), which was reported to be involved in cotton resistance to *V. dahliae* (He *et al*., 2018). Furthermore, we observed that BIN2 homologs BIL1 and BIL2 interacted with JAZ proteins, suggesting that BIN2, BIL1, and BIL2 function redundantly in interaction with JAZ proteins (Figure S3C). To further identify the functional domain responsible for their interaction, we made truncated versions of JAZ1 protein in yeast (Figure 5b). The N‐terminal domain (NT), the zinc‐finger expressed in inflorescence meristem domain (ZIM), and the jasmonate‐associated domain (Jas) of JAZ1 protein were individually deleted (Pauwels and Goossens, 2011; Wager and Browse, 2012). Deletion of the NT and Jas domains of JAZ1 did not eliminate the interaction between BIN2 and JAZ1, whereas deletion of the ZIM domain compromised their interaction, suggesting that the ZIM domain of JAZ1 is responsible for that interaction (Figure 5b).

We performed bimolecular fluorescence complementation (BiFC) assays in *Nicotiana benthamiana* leaves to further confirm the interaction between BIN2 and JAZ1 *in vivo* (Figure 5c). BIN2 was fused to an N‐terminal yellow fluorescent protein fragment (nYFP), and JAZ1 was fused to a C‐terminal YFP fragment (cYFP). Neither BIN2‐nYFP nor JAZ1‐cYFP co‐transformation with the control vectors showed any fluorescence signal. When BIN2‐nYFP and JAZ1‐cYFP were co‐expressed in *N. benthamiana* leaves, a bright fluorescence signal was observed in the cell nucleus, indicating that the interaction between BIN2 and JAZ1 occurred there. Additionally, we performed a GST pull‐down assay to confirm their interaction *in vitro* (Figure 5d). We used GST agarose to precipitate GST or BIN2‐GST proteins, and then incubated these GST proteins with purified His‐JAZ1 proteins. Recombinant His‐JAZ1 protein bound to GST‐BIN2, but not to GST. These results indicate that BIN2 physically interacts with JAZ proteins both *in vivo* and *in vitro*, and this interaction occurs at the cell nucleus.

### BIN2 phosphorylates JAZ proteins

**Figure 6:**
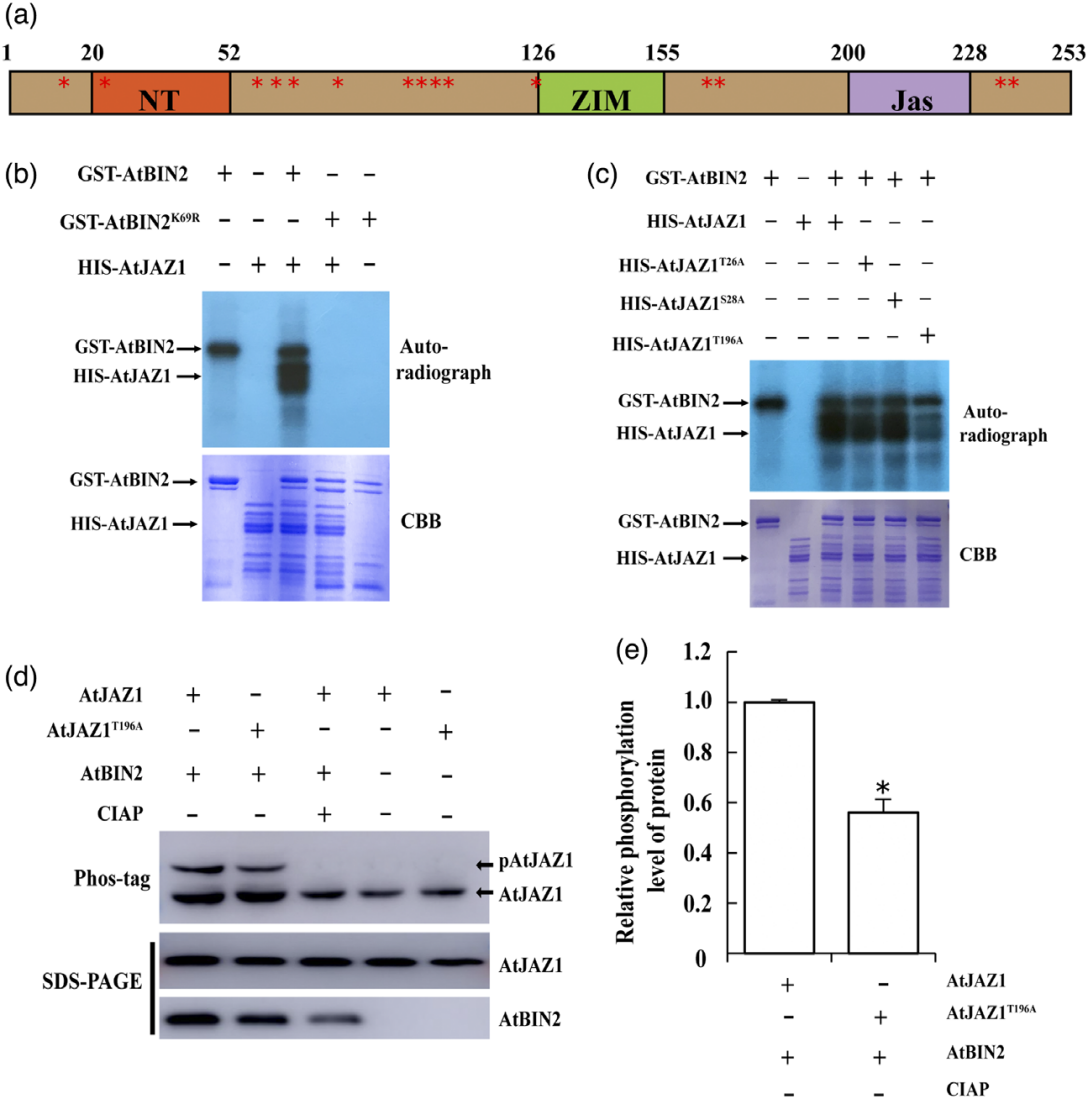
Identification of the phosphorylation residues of JAZ1 by BIN2. (a) Conserved putative BIN2 phosphorylation sites in JAZ1. (b) BIN2 phosphorylates JAZ1 *in vitro*. Recombinant His‐JAZ1 proteins were incubated with BIN2 and its kinase‐dead form BIN2^K69R^. Recombinant proteins were separated by 10% SDS‐PAGE after incubation in protein kinase buffer containing [γ‐^32^P]ATP. Phosphorylated JAZ1 was detected by autoradiography after gel electrophoresis (above panel). BIN2 plus His‐tag proteins were used as a control. Recombinant BIN2, BIN2^K69R^, and JAZ1 were detected by CBB staining (below panel). (c) *In vitro* phosphorylation assays of the phosphorylation of JAZ1 and its mutated forms by BIN2. Recombinant His‐JAZ1 and its mutated forms were incubated with BIN2. Phosphorylated JAZ1 and Recombinant proteins were detected by autoradiograph (above) and CBB‐stained gel (below), respectively. Thr‐196 on JAZ1 is the BIN2 phosphorylation site. (d) Phos‐tag assays showing the phosphorylation status of JAZ1 and JAZ1^T196A^ *in vivo*. Plasmids of the indicated combinations were transformed into *N. benthamiana* protoplasts and expressed at 22 °C for 12 h. Protein extracts from the protoplasts were separated in a Phos‐tag gel, and MYC‐JAZ1was then detected with anti‐MYC antibody (upper panel). The levels of JAZ1 (middle, anti‐MYC) and BIN2 (bottom, anti‐FLAG) proteins are shown in the SDS‐PAGE gels. Three independent experiments showed consistent results. (e) The relative phosphorylation level of JAZ1 and JAZ1^T196A^ in (d). Relative levels of AtJAZ1 were defined as “1”. Error bars represent ± SD (*n *= 3). Asterisks (*P < 0.05) indicate statistically significant differences.

As a kinase, BIN2 can phosphorylate most of the proteins with which it interacts (Youn and Kim, 2015). We further investigated whether JAZ1 was a substrate for phosphorylation by BIN2. S/TxxxS/T (S/T corresponds to Ser or Thr and x denotes any other residue) is the conserved phosphorylation motif targeted by BIN2 (Ryu *et al*., 2010; Ryu *et al*., 2007; Wang *et al*., 2002; Woodgett, 2001). Bioinformatic analysis indicated that several putative BIN2 phosphorylation sites were identified in the JAZ1 protein sequence, suggesting that JAZ1 may be a substrate of BIN2 (Figure 6a). To verify the phosphorylation of JAZ1 by BIN2, we conducted *in vitro* phosphorylation assays (Figure 6b). These revealed that JAZ1 was specifically phosphorylated by BIN2 *in vitro*. Conversely, a kinase‐dead mutant BIN2^K69R^ failed to phosphorylate JAZ1 protein (Figure 6b; Li and Nam, 2002). To identify the BIN2‐mediated potential phosphorylation sites of JAZ1, we performed mass spectrometry analysis and found three potential phosphorylation sites T26, S28, T196 in JAZ1 protein (Figure S4). To determine whether these amino acids of JAZ1 are phosphorylated by BIN2, we mutated these residues into alanine to make single‐mutated forms of JAZ1 and performed *in vitro* kinase assays. JAZ1 and all the mutated forms were phosphorylated by BIN2; however, the phosphorylation levels of JAZ1^T196A^ by BIN2 were significantly reduced (Figure 6c). These results indicate that JAZ1^T196^ is a major phosphorylated site targeted by BIN2, while the other two sites are not. To investigate whether JAZ1 is phosphorylated *in vivo*, we expressed MYC‐JAZ1/HF‐BIN2 or MYC‐JAZ1 alone in *N. benthamiana* protoplasts and performed phos‐tag mobility shift assays. As shown in Figure 6d, a slow‐migrating form of JAZ1 was detected and eliminated after incubating the protein samples with calf intestinal alkaline phosphatase (CIAP), suggesting that the band corresponded to phosphorylated JAZ1. These results indicate that JAZ1 is phosphorylated by BIN2 in planta. We also examined the effect of BIN2 on the phosphorylation of the mutated form MYC‐JAZ1^T196A^. We found that although the mutated form JAZ1^T196A^ was still phosphorylated by BIN2, the phosphorylation level was considerably reduced as compared with wild‐type JAZ1 protein (Figure 6d and e). These results demonstrate that JAZ1^T196A^ is a major phosphorylation site of BIN2.

### BIN2 promotes the degradation of the JAZ1 protein

**Figure 7:**
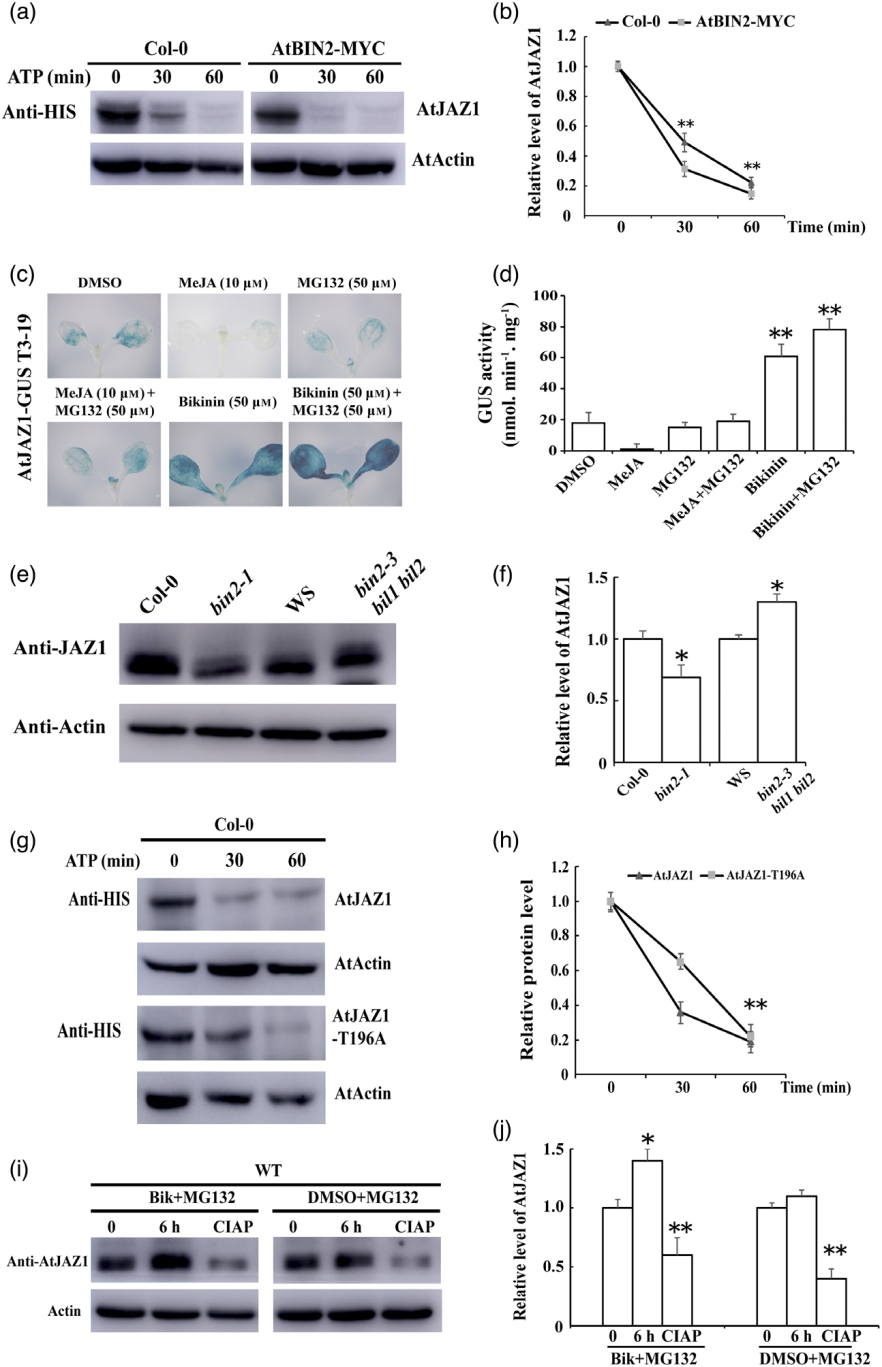
BIN2 destabilizes JAZ1 through phosphorylation. (a) and (b) BIN2 promotes JAZ1 degradation in a cell‐free degradation assay. The wild‐type *Col‐0* and BIN2‐MYC overexpression plants were grown on MS for 14 days. Equal amounts of total proteins were extracted from these plants and combined with recombinant His‐JAZ1 and 10 mm ATP. Anti‐His antibody was used to detect JAZ1, and Actin was used as the internal control. Relative levels of AtJAZ1 at 0 h were defined as “1.” (c) and (d) *35S‐AtJAZ1‐GUS* T3‐19 overexpression transgenic lines were treated with DMSO, MeJA (10 μm), MG132 (50 μm), MeJA (10 μm) + MG132 (50 μm), Bikinin (50 μm), or Bikinin (50 μm) + MG132 (50 μm) for 48 h. The leaves were used to perform the GUS staining assays. GUS activity quantification of *35S‐AtJAZ1‐GUS* T3‐19 overexpression transgenic lines was performed. (e) and (f) The protein levels of AtJAZ1 in WT and BIN2‐related mutants. The AtJAZ1 protein was detected with anti‐AtJAZ1 antibody and actin was used as a loading control. (g) and (h) The mutant protein JAZ1‐T196A is more stable than JAZ1 in a cell‐free degradation assay. Equal amounts of the recombinant proteins AtJAZ1‐His and AtJAZ1‐T196A‐His were incubated with equal amounts of total proteins extracted from 14‐day‐old Col‐0 wildtype plants in the *in vitro* cell‐free degradation assays. Anti‐His antibody was used to detect recombinant proteins, and actin was used as the internal control. Relative levels of AtJAZ1 and AtJAZ1‐T196A at 0 h were defined as “1”. (i) and (j) The degradation of AtJAZ1 is related with BIN2‐mediated phosphorylation *in vivo*. 14‐day‐old Col‐0 wild‐type plants were treated with indicated combinations for different hours. Anti‐AtJAZ1 antibody was used to detect JAZ1 proteins, and actin was used as the internal control. CIAP represents Calf Intestinal Alkaline Phosphatase.

Previous studies have shown that BIN2 phosphorylates and regulates the stability of its substrates in plants (Cheng *et al*., 2014; Gudesblat *et al*., 2012; He *et al*., 2002; Vert *et al*., 2008; Ye *et al*., 2019; Yin *et al*., 2002). To investigate whether BIN2 regulates the stability of JAZ1, we conducted cell‐free protein degradation assays (Figure 7a and b). His‐JAZ1 recombinant proteins were incubated with an equal amount of total proteins extracted from *Col‐0* and BIN2‐overexpressing transgenic plants (AtBIN2‐MYC) supplemented with ATP. Interestingly, we found that the degradation rates of the JAZ1 protein in *Col‐0* were much slower than in BIN2‐overexpressing transgenic plants. These results suggest that BIN2 destabilizes JAZ1 *in vitro*. Besides, we used the GUS (β‐glucuronidase) reporter system to examine the stability of JAZ1 protein under BIN2 inhibition (Jefferson *et al*., 1987). 35S‐*JAZ1‐GUS* transgenic plants were generated and exposed to MeJA and bikinin (Figures 7c, d, and S5). MeJA promoted the degradation of JAZ1 protein, while bikinin treatment induced a significant increase in GUS activity in these transgenic lines. Besides, we examined the protein levels of JAZ1 in BIN2‐related mutants. We found that the levels of JAZ1 were obviously lower in the *bin2‐1* mutants but more in the *bin2‐3 bil1bil2* mutants than those in the corresponding wildtype plants (Figure 7e and f). These observations further confirm that BIN2 promotes the degradation of the JAZ1 protein *in vivo*.

Furthermore, cell‐free protein degradation assays revealed that JAZ1^T196A^ was more stable than JAZ1 when incubated with total proteins extracted from *Col‐0* plants (Figures 7g and h). As indicated in Figure 7i and j, the accumulation of JAZ1 protein was increased under the treatment of bikinin and can be partly eliminated by CIAP treatment. These results indicate that the phosphorylation mediated by BIN2 is required for JAZ1 degradation.

### Phosphorylation of JAZ1 mediated by BIN2 influences JA transduction

**Figure 8:**
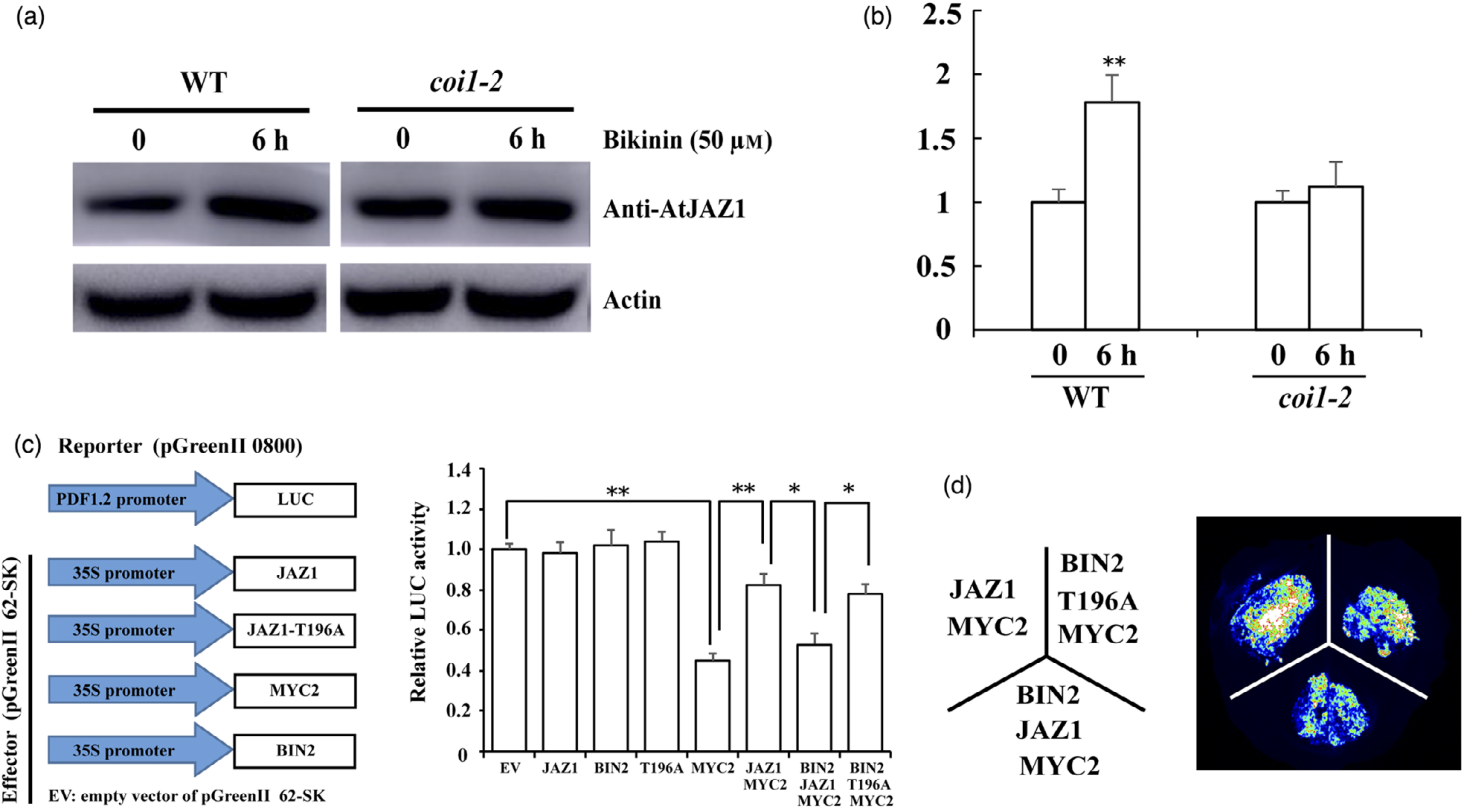
AtBIN2‐AtJAZ1 interaction affects JA pathway transduction. (a) and (b) Accumulation of AtJAZ1 induced by Bikinin was inhibited in *coi1‐2* mutants. 14‐day‐old Col‐0 and *coi1‐2* mutants were treated with 50 μm Bikinin for 6 h. Total proteins were extracted at different times after treatments and were detected by anti‐AtJAZ1 antibody. Actin was used as the internal control. (c) and (d) BIN2 attenuates JAZ1 inhibition on MYC2‐mediated repression of *PDF1.2* expression. The reporter construct p*PDF1.2*‐LUC with the indicated effector constructs shown in (c) were co‐transformed into *N. benthamiana* leaves for 72 h; leaves transformed with p*PDF1.2*‐LUC and empty vector pGreenII 62‐SK were used as a control. Relative LUC activity indicated the *PDF1.2* expression level; the control value was set to 1.0. LUC activity indicates the accumulation level of *PDF1.2* with JAZ1 or JAZ1‐T196A in *N. benthamiana* leaves. Error bars represent ± SD (*n* = 3). Each independent experiment contains at least 10 plants per treatment. Asterisks (*P < 0.05, **P < 0.01) indicate statistically significant differences.

Considering JAZ protein is a negative regulator of the JA signalling pathway, we investigated whether BIN2 phosphorylation affects JA signalling transduction. The *coi1‐2* mutants are leaky mutant alleles of JA signal receptor COI1, which exhibited reduced JA insensitivity and partial fertility (Xu *et al*., 2002). We found that the bikinin‐mediated stabilization of AtJAZ1 was inhibited in *coi1‐2* mutants (Figure 8a and b), suggesting the involvement of AtCOI1 in AtBIN2‐mediated AtJAZ1 stability.

*PDF1.2* is a JA‐responsive gene that contributes to plant response to pathogens and is negatively regulated by the JA signal transcription factor *MYC2* (Dombrecht *et al*., 2007; Lorenzo *et al*., 2004). *PDF1.2* was therefore used as a target to assess the roles of BIN2 by using a LUC assay *in vivo* (Figures 8c and d). LUC activity was lower in plants transformed with *MYC2* as compared with the empty vector control, suggesting that *MYC2* indeed inactivates *PDF1.2* expression. The inhibition effect of *PDF1.2* imposed by *MYC2* was significantly attenuated by the addition of *JAZ1*. When *BIN2* was co‐expressed with *JAZ1* and *MYC2*, the fluorescence signal was lower than in the plants injected with *JAZ1* and *MYC2*. Furthermore, plants injected with *JAZ1^T196A^*, *BIN2*, and *MYC2* induced LUC activity more strongly than plants injected with *JAZ1*, *BIN2*, and *MYC2*. These results suggest that the phosphorylation of JAZ1 by BIN2 influences JA signalling transduction.

## Discussion

Cotton is the most important natural fibre crop in the world, widely cultivated in almost 150 countries and integral in economical production worldwide (Zhang *et al*., 2018). However, cotton yield and quality are significantly reduced by cotton *Verticillium wilt*, which is also called “cancer of cotton”. This destructive disease causes massive annual losses in cotton crop yields (Gong *et al*., 2018; Wang *et al*., 2016). Many studies have suggested that the BR signalling pathway is involved in plant resistance to *V. dahliae* (Anwar *et al*., 2018; Gao *et al*., 2013; Nawaz *et al*., 2017; Wang, 2012; Yu *et al*., 2018). BIN2 is a negative regulator of the BR signalling pathway, and the expression level of BIN2 was significantly decreased in *Gossypium barbadense* after *V. dahliae* infestation (Gao *et al*., 2013). However, the function and molecular mechanism of BIN2 in plant resistance to *V. dahliae* were poorly understood.

In the current study, we revealed that transgenic cotton lines constitutively expressing *GhBIN2* and AtBIN2 gain‐of‐function *Arabidopsis* mutants *bin2‐1* exhibited much more severe symptoms after *V. dahliae* infestation than did the control plants (Figures 3 and 4), while the *GhBIN2*‐silenced plants and AtBIN2 loss‐of‐function mutants *bin2‐3 bil1 bil2* showed increased resistance capability against *V. dahliae* (Figures 2 and 4). These observations confirmed that BIN2 functioned as a negative regulator of plant defence against *V. dahliae* in both *Arabidopsis* and cotton. In previous study, overexpression of MsK1, a GSK from *Medicago sativa*, was demonstrated to increase susceptibility to the virulent bacterial pathogen *Pseudomonas* and to compromise MAP kinase activation with pathogen infection (Wrzaczek *et al*., 2007). Recent work revealed that GSK3 enhanced antiviral defence in rice (He *et al*., 2020). Therefore, we propose that BIN2 may feature prominently in the plant response to various pathogens. Further, *BIN2* may be used as a candidate gene for generating disease‐resistant crops by using the CRISPR/Cas9 genome editing system or RNAi approaches.

In *Arabidopsis*, BIN2 has two homologs, BIL1 and BIL2, which function redundantly (Jonak and Hirt, 2002). In cotton, analysis of the *GSK* gene family found six *BIN2* homologous genes in *G. hirsutum* including *GhSK21*, *GhSK22*, *GhSK23*, *GhSK24*, *GhSK25*, and *GhSK26* (Wang *et al*., 2018). GhSK24 corresponds to GhBIN2 in the current study (Table S1). GhBIN2 and the other five homologs existed high sequence similarities. This functional redundancy mediated by additional homologs may affect the effectiveness of genetic engineering, which should be taken into consideration when GhBIN2 is the target of RNA silencing or genetic editing in the crops. BIN2 is the first plant GSK3‐like kinase characterized from genetic screening and negatively influences BR signalling to regulate plant growth and development (Li and Nam, 2002; Li *et al*., 2001). Editing BIN2 could generate undesired effects on growth and development in plants, but the available evidence shows that BIN2 silenced *Arabidopsis* plants show better growth performance than that of control plants (Xiong *et al*., 2017), suggesting that BIN2 may play a role in balancing plant growth and defence.

In this study, two‐hybrid screening, *in vivo* BiFC, and pull‐down assays indicated that BIN2 interacted with twelve of the thirteen JAZ proteins and that the ZIM domain of the JAZ1 protein was responsible for their interactions (Figure 5). Previous studies showed that the ZIM domain acted as a protein–protein interaction domain, which mediated homo‐ and heteromeric interactions between JAZ proteins as well as the interaction between JAZ and NINJA (Chini *et al*., 2009). The interaction between BIN2 and JAZ proteins may influence the function of JAZ dimerization and interaction with NINJA. GSK2 was recently reported to influence the JAZ4‐NINJA complex and JAZ4‐JAZ11 dimerization in the monocotyledon rice (He *et al*., 2020). Further experiments are required in dicotyledons, including cotton and *Arabidopsis*. Subsequent experiments showed that BIN2 could phosphorylate JAZ. Mass spectrometry, *in vitro* phosphorylation assays and phos‐tag mobility shift assays showed that T196 was the phosphorylation site mediated by BIN2, and was required for this regulation (Figures S4, 6 and 7). In apple (*Malus domestica*), MdSnRK1.1 was integral to sucrose‐induced biosynthesis of anthocyanins due to its interaction with and phosphorylation of MdJAZ18 protein (Liu *et al*., 2017a). In our study, we showed that BIN2 could act as a new upstream protein kinase to phosphorylate JAZ proteins. This observation reveals that BIN2 facilitates the interaction between the BR and JA signalling pathways.

BIN2 phosphorylation plays dual roles in regulating the stability and activity of its substrates. BIN2 phosphorylates and destabilizes its substrates or inhibits their activities, including BES1, BZR1, SPCH (SPECHESS), EGL3 (ENHANCER OF GLABRA3), TTG1 (TRANSPARENT TESTA GLABRA1), ARF2 (AUXIN RESPONSE FACTOR2), ICE1 (INDUCER OF CBF EXPRESSION1) (Cheng *et al*., 2014; Gudesblat *et al*., 2012; He *et al*., 2002; Vert *et al*., 2008; Ye *et al*., 2019; Yin *et al*., 2002). BIN2 also positively regulates several proteins (Cai *et al*., 2014; Cho *et al*., 2014; Hu and Yu, 2014; Ye *et al*., 2012; Zhang *et al*., 2014). In this study, we revealed that BIN2 destabilized the JAZ protein by direct phosphorylation, and affected JA signalling transduction (Figures 7 and 8). Previous studies reported that SA played a crucial role in the activation of defence responses against biotrophic and hemibiotrophic pathogens (including *V. dahliae*) and that the SA and JA defence pathways were mutually antagonistic (Beckers and Spoel, 2006; Grant and Lamb, 2006; Mur *et al*., 2006). We propose that the JAZ protein phosphorylated by BIN2 activates the JA signalling pathway and thus inhibits the SA defence pathways, which enhances plant susceptibility to *V. dahliae*. In previous study, researchers have revealed that JA mutants like *jar1*, *coi1‐16* and *cyp94B3* showed less severe disease symptom development than that of the WT control (Scholz *et al*., 2018). These observations were consistent with our results, showing that the activated JA signalling pathway enhanced plant susceptibility to *V. dahliae*. Taken together, our results demonstrate the negative role of BIN2 in plant defence against *V. dahliae*, and advance understanding of the interaction between BR and JA signalling. In the future study, more evidence is needed to clarify whether JA and SA antagonism is essential for BIN2‐regulated the resistance to *V. dahliae*.

## Methods

### Plant materials and culture conditions

The *Arabidopsis* mutant lines *bin2‐1*, *bin2‐3*, *bin2‐3 bil1 bil2*, and BIN2 overexpression transgenic lines were kindly provided by Dr. Jianming Li of Shanghai Institutes of Biological Sciences, Chinese Academy of Sciences, and Dr. Shuhua Yang of College of Biological Sciences, China Agricultural University. The *Arabidopsis* seeds were sterilized according to established methods (Song *et al*., 2017). *Arabidopsis thaliana* plants were grown at 22 °C under long‐light conditions (16 h light/8 h dark) on 1/2 Murashige and Skoog (MS) medium plates containing 0.8% agar. WT *Gossypium hirsutum* (ZM24) was used in this study, and the cotton plants were grown in a growth chamber at 25 °C under 16 h light/8 h dark conditions. The seeds of tobacco *Nicotiana benthamiana* were sown in soil and grown in a greenhouse under the conditions described in a previous study (Wu *et al*., 2012).

### Quantitative real‐time PCR (QRT‐PCR)

Total RNA was extracted from *Arabidopsis* or cotton seedlings using the RNAprep Pure Plant kit (Tiangen, Beijing, China), followed by first‐strand cDNA product generation using the PrimeScript RT reagent kit (Takara, Beijing, China). The relative transcript levels were measured using SYBR Premix Ex Taq™ (Tli RNase H Plus) (Takara) and ABI 7900 qRT‐PCR System (Applied Biosystems, Foster City, CA). Expression levels of the target genes were normalized to *AtACTIN2* in *Arabidopsis* or *GhHISTONE3* in cotton. The 2^−ΔΔCt^ method was applied to calculate the relative expression level of all target genes (Livak and Schmittgen, 2001). The specific primers used in this study were designed by Primer Premier version 5.0 (Table S2).

### Virus‐induced gene silencing (VIGS) experiments

The conserved cDNA fragments of *GhBIN2* (GenBank No.: KM453729) were amplified from the cDNA of *G. hirsutum* (ZM24), and the corresponding primer pairs used were found in Table S2 (Liu *et al*., 2002). We constructed the pTRV2 vector that contains the coding sequence of *GhBIN2* to suppress its expression. The pTRV2 vector (TRV:00) was used as a negative control, and the pTRV2 vector with the cDNA of *GhPDS* was used as a positive control to indicate the VIGS efficiency. The amplified fragments were cloned into the TRV2 plasmid at the EcoRI‐BamHI sites using the In‐Fusion HD Cloning Kit (Clontech) according to the manufacturers’ protocol. The pTRV1, pTRV2, and pTRV2 derivatives harboring the cDNA fragments of target genes were transformed into *Agrobacterium tumefaciens* GV3101. Cotton VIGS experiments were performed following established procedures (Pang *et al*., 2013; Song *et al*., 2018). The vectors were agro‐infiltrated into the cotyledons of 7‐day‐old cotton seedlings. Two weeks after infiltration, RNA was extracted from these cotton seedlings to measure the expression of the target genes.

### Preparation and inoculation of fungal pathogens

The antagonistic defoliating Vd07038 isolate of *V. dahliae* was used in this study. The fungal strain was cultured on potato dextrose agar (PDA) medium at 25 °C for 7 days. Next, the colonies were transformed into Czapek medium (3% (w/v) sucrose, 0.2% (w/v) NaNO3, 0.131% (w/v) K2HPO4, 0.05% (w/v) KCl, 0.05% (w/v) MgSO4, and 0.002% (w/v) FeSO4), and inoculated on a shaker at 200 rpm at 25 °C for 7 days. The conidial suspensions were adjusted with deionized water to a final spore concentration of 10^7^ ML^−1^ for cotton. To inoculate the *Arabidopsis*, conidial suspensions were diluted with distilled water to 5 × 10^3^ mL^−1^, and drops (2 μL) were used to inoculate the roots of 2‐week‐old seedlings (Gong *et al*., 2018). The rate of diseased cotton and the disease index (DI) was calculated as previously described (Gong *et al*., 2018). For *Arabidopsis*, the DI, the extent of stunting and leaf chlorosis were calculated according to the formulas described previously (Veronese *et al*., 2003).

### Reactive oxygen species (ROS) detection in cotton leaves

After the cotton seedlings were inoculated with *V*. *dahliae* for 24 h, cotton leaves were collected and washed using distilled water. The production and accumulation of ROS species were detected using the 3,3′‐Diaminobenzidine (DAB) staining method as previously described (Reissig *et al*., 1955). An optical microscope (Nikon, Tokyo, Japan) was used to observe and photograph the stained ROS patches.

### *Verticillium dahliae* recovery assay

To identify the effect of infection by *V. dahliae*, cotton stem fragments (4.5 cm) from the first stem node at 20 days post‐inoculation (dpi) were analysed. These stem segments were surface‐sterilized with 75% ethanol for 1 min, followed by 5% NaClO for 45 s, and then rinsed with sterile water three times. These fragments were sliced into five parts, placed on potato dextrose agar plates, and incubated at 25 °C. Each experiment was carried out using three biological replicates.

### Two‐hybrid screening

The full‐length encoding sequences of *AtBIN2*, *AtBIL1*, *AtBIL2*, and *GhBIN2* were cloned into the bait vector pGBKT7. The full‐length cDNA sequences of *AtJAZ*s, *AtOPR3*, *AtJAR1*, *AtCOI1*, *AtNINJA*, *AtMYC2*, and *GhJAZ2* were fused into the prey vector pGADT7. These constructs were transformed into the yeast strain Y2HGold (Clontech) and grown on SD‐Leu‐Trp (‐LW) plates. The yeast transformants were screened on SD‐Leu‐Trp‐His‐Ade (‐LWHA) selective medium. Primers used for constructing various clones in this study are listed in Table S2.

### Bimolecular fluorescence complementation (BiFC) assays

The full‐length cDNA sequences of *AtBIN2* were amplified and cloned into pUC‐SPYNE to generate an N‐terminal in‐frame fusion with nYFP. Full‐length *AtJAZ1* encoding sequences were inserted into pUC‐SPYCE to generate a C‐terminal in‐frame fusion with cYFP. The corresponding primer pairs used are shown in Table S2. The resulting vectors were introduced into *Agrobacterium tumefaciens* strain GV3101 and transformed into *N. benthamiana* plants. Infected leaves were analysed 48 h after infiltration using a confocal microscope (Olympus FV1200).

### Purification of recombinant proteins and pull‐down assays

AtBIN2 full‐length cDNA was cloned into the pGEX4T‐1 vector with the GST‐tag, and AtJAZ1 full‐length cDNA was cloned into the pET32a vector with the His‐tag. The resulting plasmids were transformed into *E. coli* strain BL21 (DE3). The recombinant proteins AtBIN2‐GST and AtJAZ1‐His were purified with Glutathione Sepharose™ 4 Fast Flow and Ni Sepharose™ 6 Fast Flow (GE Healthcare, Pittsburgh, PA), respectively, according to the manufacturers’ instructions. The pull‐down assays were performed as described in previous studies (Wang *et al*., 2011). Proteins retained on the beads were analysed by immunoblotting with an anti‐GST or anti‐His antibody.

### *In vitro* phosphorylation assay

The *in vitro* phosphorylation assay was performed according to standard methods (Ding *et al*., 2015). Purified recombinant protein combinations were incubated in protein kinase assay buffer with 20 mm Tris‐HCl pH 7.5, 10 mm MgCl_2_, 100 mm NaCl, 25 mm ATP, 1 µCi [γ‐^32^P] ATP, and 1 mm DTT at 30 °C for 30 min. The reactions were halted by the addition of a 5× SDS loading buffer. The phosphorylated protein was visualized by autoradiography after separation in a 10% SDS‐PAGE gel and detected by a Typhoon 9410 imager. Coomassie brilliant blue was used as a loading control.

### Identification of phosphorylation site

To identify the putative phosphorylation sites of BIN2 in JAZ1, we performed LC‐MS/MS experiments. Purified GST‐BIN2 and His‐JAZ1 proteins were incubated in 20 μL of protein kinase assay buffer (20 mm Tris‐HCl pH 7.5, 10 mm MgCl_2_, 100 mm NaCl, 25 mm ATP, and 1 mm DTT) at 30 °C for 30 min. The reaction mixture was reduced by DTT, alkylated by iodoacetamide (IAM), and digested by trypsin overnight at 37 °C. The Titansphere Phos‐TiO Kit (GL Sciences) was used to enrich the phosphopeptides according to the manufacturer’s protocol. The resulting phosphopeptides were analysed by LC‐MS/MS, performed as previously described (Liu *et al*., 2017b).

### Phos‐tag mobility shift assay

The Phos‐tag mobility shift assay was performed as described previously (Mao *et al*., 2011). Total proteins extracted from protoplasts were separated in a 12% SDS‐PAGE gel containing 50 mm Phos‐tag and 200 mm MnCl_2_. After electrophoresis, the gel was washed three times in transfer buffer (50 mm Tris, 40 mm Glycine) for 10 min each time. Then, the gel was transferred to a polyvinylidene fluoride membrane. The AtJAZ1‐MYC was detected with the anti‐MYC antibody. Anti‐FLAG antibody was used to detect AtBIN2‐HF. The CIAP treatments were conducted by adding CIAP with its reaction buffer to total proteins as described by the manufacturer before SDS‐PAGE. The integrated optical density (IOD) values of the indicated signals were quantified using the software Image‐Pro Plus (Media Cybernetics).

### JA content measurements

Two‐week‐old *Arabidopsis* seedlings were harvested, and measurements of JA contents were performed as described previously (Song *et al*., 2017). Three biological experiments which consisted of 10 plants per experiment were measured.

### GUS staining and GUS activity quantification

The GUS staining and activity quantification assay was performed as described previously (Menand *et al*., 2002). Images were captured using a stereomicroscope (OLYMPUS MVX10). We used a MarkerGene™ β‐glucuronidase (GUS) reporter gene activity detection kit (Marker Gene Technologies) to quantify GUS activity. The Bradford assay was used for total protein quantification. Three biological experiments, each consisting of 20 plants per treatment, were measured.

### Cell‐free protein degradation assay

The cell‐free protein degradation assay was performed as described previously (Liu *et al*., 2010). Total proteins were extracted from 14‐day‐old seedlings in a degradation buffer (50 mm Tris‐MES pH 8.0, 0.5 M Sucrose, 1 mm MgCl_2_, 10 mm EDTA pH 8.0, and 5 mm DTT). Purified recombinant His‐JAZ1 or His‐JAZ1^T196A^ was added to an equal amount of the indicated proteins and incubated at 25 °C for 0, 30, and 60 min. His‐JAZ1 or His‐JAZ1^T196A^ was separated on SDS‐PAGE and detected with the anti‐His antibody. The integrated optical density (IOD) values of the indicated signals were quantified using the software Image‐Pro Plus (Media Cybernetics).

### Dual‐luciferase reporter assays

The transient dual‐luciferase reporter assays were performed as described previously (Hellens *et al*., 2005). The promoter sequences of *AtPDF1.2* were amplified and cloned into pGreenII 0800‐LUC at the PstI and SpeI sites. The coding regions of AtMYC2, AtJAZ1, AtJAZ1^T196A^, and AtBIN2 were amplified and cloned into pGreenII 62‐SK at the SpeI and EcoRI sites. Vectors were co‐transformed into 4‐weeks‐old *N. benthamiana* leaves and expressed for 72 h, and the images were captured by CCD. These leaves were collected and grounded in liquid nitrogen. Firefly luciferase and *Renilla spp*. luciferase activities were quantified using the Dual‐Luciferase Reporter Assay System (Promega) with a multimode plate reader (PerkinElmer, Waltham, MA).

### Antibody validation

Immunoblotting was performed to detect the polyclonal antibody anti‐AtJAZ1 (Agrisesera, Sweden). The wildtype *Col‐0*, AtJAZ1‐overexpression transgenic lines and *jaz1* mutant were used for protein extraction, and one specific band for this antibody was detected within the total protein fraction of plants tested (Figure S6).

## Conflict of interest statement

Authors confirm no conflict of interests to declare.

## Author contributions

FL, MR, and YS designed the experiments. YS, YZ, LL, ZY, and YG performed the experiments. CZ and ZY analysed the data. MR and YS wrote the manuscript.

## Supporting information

**Figure S1** Disease index assessment of *GhBIN2* over‐expression transgenic lines.
**Figure S2** Phenotypes of *A. thaliana* BIN2 mutants in defense response to *V. dahliae*.
**Figure S3** BIN2, BIL1, and BIL2 function redundantly in interactions with JAZ proteins.
**Figure S4** Mass spectrometry analysis of BIN2 phosphorylation sites in JAZ1.
**Figure S5** BIN2 induces the degradation of AtJAZ1.
**Figure S6** Validation of AtJAZ1 antibody.

**Table S1** Characteristics of *BIN2* genes in the cotton genome.

**Table S2** Primers used in this study.
