# Supplementary material for: BIN2 negatively regulates plant defence against Verticillium dahliae in Arabidopsis and cotton
Source: Plant Biotechnol J. 2021 Jun 11;19(10):2097–112. doi: 10.1111/pbi.13640 (PMC8486250; doi:10.1111/pbi.13640)
Supplement: Supplementary file 1 — Figure S1 Disease index assessment of GhBIN2 over‐expression transgenic lines. Figure S2 Phenotypes of A. thaliana BIN2 mutants in defense response to V. dahliae. Figure S3 BIN2, BIL1, and BIL2 function redundantly in interactions with JAZ proteins. Figure S4 Mass spectrometry analysis of BIN2 phosphorylation sites in JAZ1. Figure S5 BIN2 induces the degradation of AtJAZ1. Figure S6 Validation of AtJAZ1 antibody. [file PBI-19-2097-s002.docx]

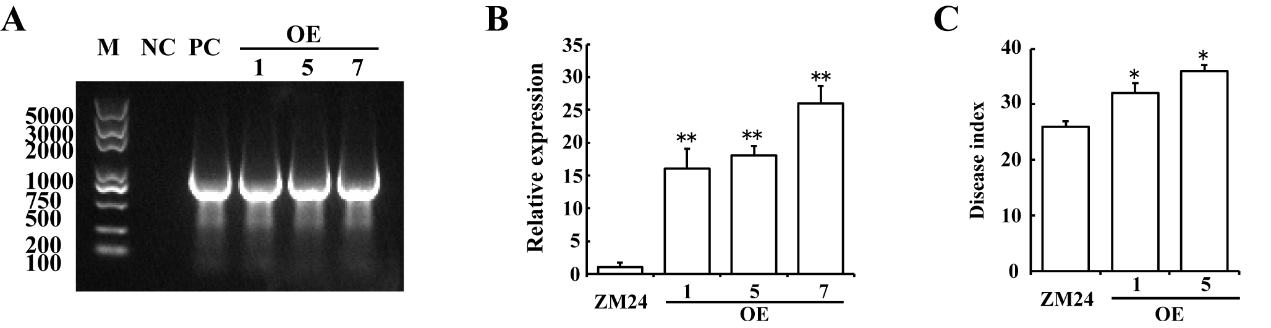


**Figure S1. Disease index assessment of *GhBIN2* over-expression transgenic lines**

(A) Identification of *GhBIN2* over-expression transgenic lines by PCR. NC represents wild-type cotton seedlings. NC: negative control; PC: positive control; OE: over-expression transgenic lines. (B) Expression levels of *GhBIN2* in transgenic cotton lines (OE1, OE5, and OE7). *GhHistone 3* was used as an internal control. (C) Disease index of the ZM24 and *GhBIN2* overexpression transgenic plants (OE1 and OE5) at 25 dpi. Data are the mean values of three replicates ± SD. Each independent experiment contains at least 10 plants per treatment. Asterisks (**p*<0.05, ***p*<0.01) indicate statistically significant differences.


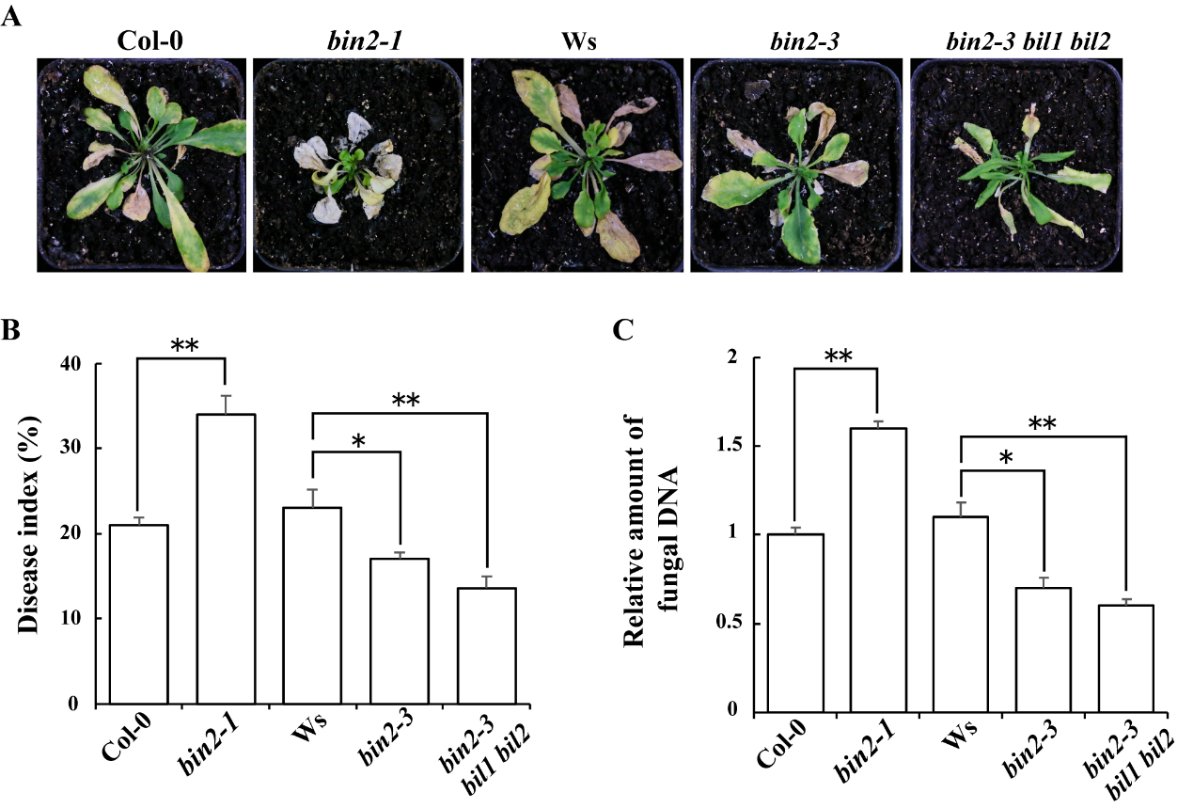


**Figure S2. Phenotypes of *A. thaliana* BIN2 mutants in defense response to *V. dahliae*.**

(A) Disease symptoms induced by *V. dahliae* strain 07038 on the rosette leaves of *Col-0*, *bin2-1*, Ws, *bin2-3*, and *bin2-3 bil1 bil2*. 3-week-old *Arabidopsis* seedlings were inoculated with *V. dahliae*, and photographs were taken at 20 dpi. (B) Assessment of disease index of *Col-0*, *bin2-1*, Ws, *bin2-3*, and *bin2-3 bil1 bil2* at 20 dpi. (C) The relative amount of fungus in *Col-0*, *bin2-1*, Ws, *bin2-3*, and *bin2-3 bil1 bil2* plants at 20 dpi. The relative amount of fungus was determined by quantitative real-time PCR. Error bars represent ±SD (n=3). Asterisks (**p*<0.05, ***p*<0.01) indicate significant differences between corresponding control and BIN2 mutants.


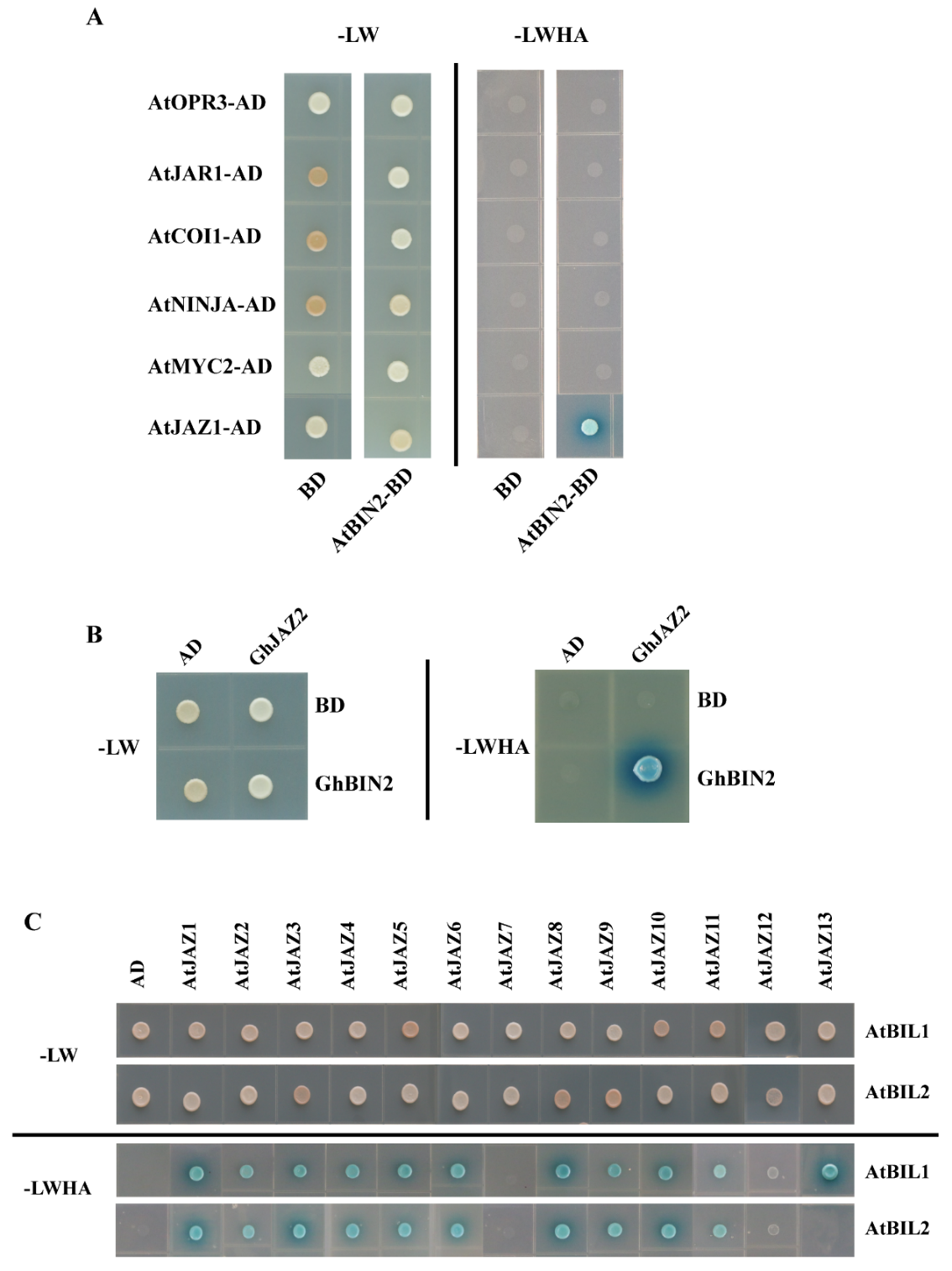


**Figure S3. BIN2, BIL1, and BIL2 function redundantly in interactions with JAZ proteins**

(A) Screening BIN2 interaction protein in the JA signaling pathway by two-hybrid assays. (B) GhBIN2 interacts with GhJAZ2 in yeast two-hybrid analysis. (C) Yeast two-hybrid analysis of AtBIL1, AtBIL2, and AtJAZ proteins. AtJAZs interact with BIL1 and BIL2 in the yeast two-hybrid system. The empty vector pGADT7 or pGBKT7 was used as the negative control. –LW represents SD-Leu-Trp plates. –LWHA represents SD-Leu-Trp-His-Ade plates.


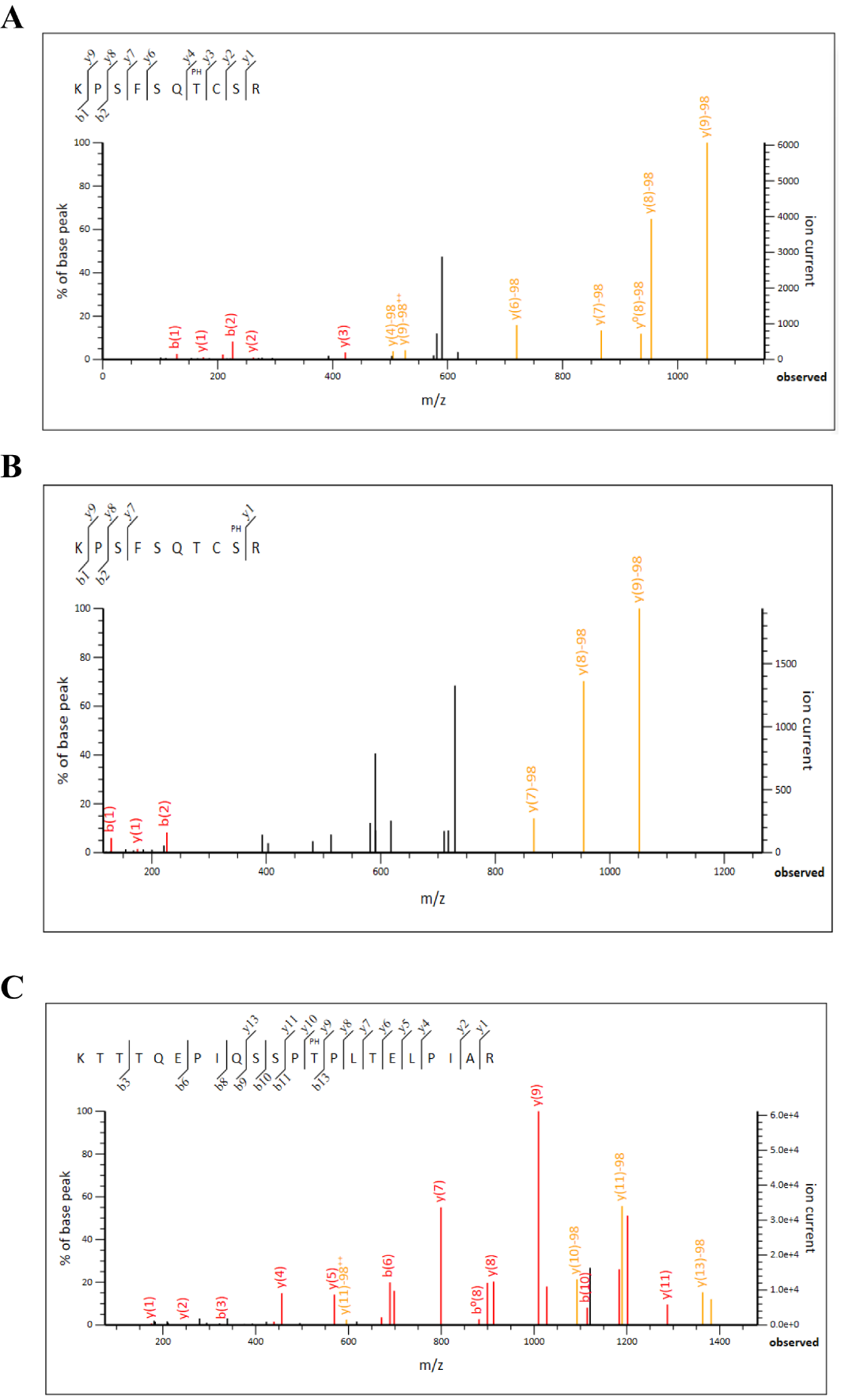


**Figure S4. Mass spectrometry analysis of BIN2 phosphorylation sites in JAZ1**

Recombinant GST-BIN2 and His-JAZ1 proteins were added to the reaction buffer and incubated at 30°C for 30 min, followed by mass spectrum assays. The mass spectrum result indicates JAZ1 Thr26 (A), Ser28 (B), Thr196 (C) are BIN2 potential phosphorylation sites in JAZ1.


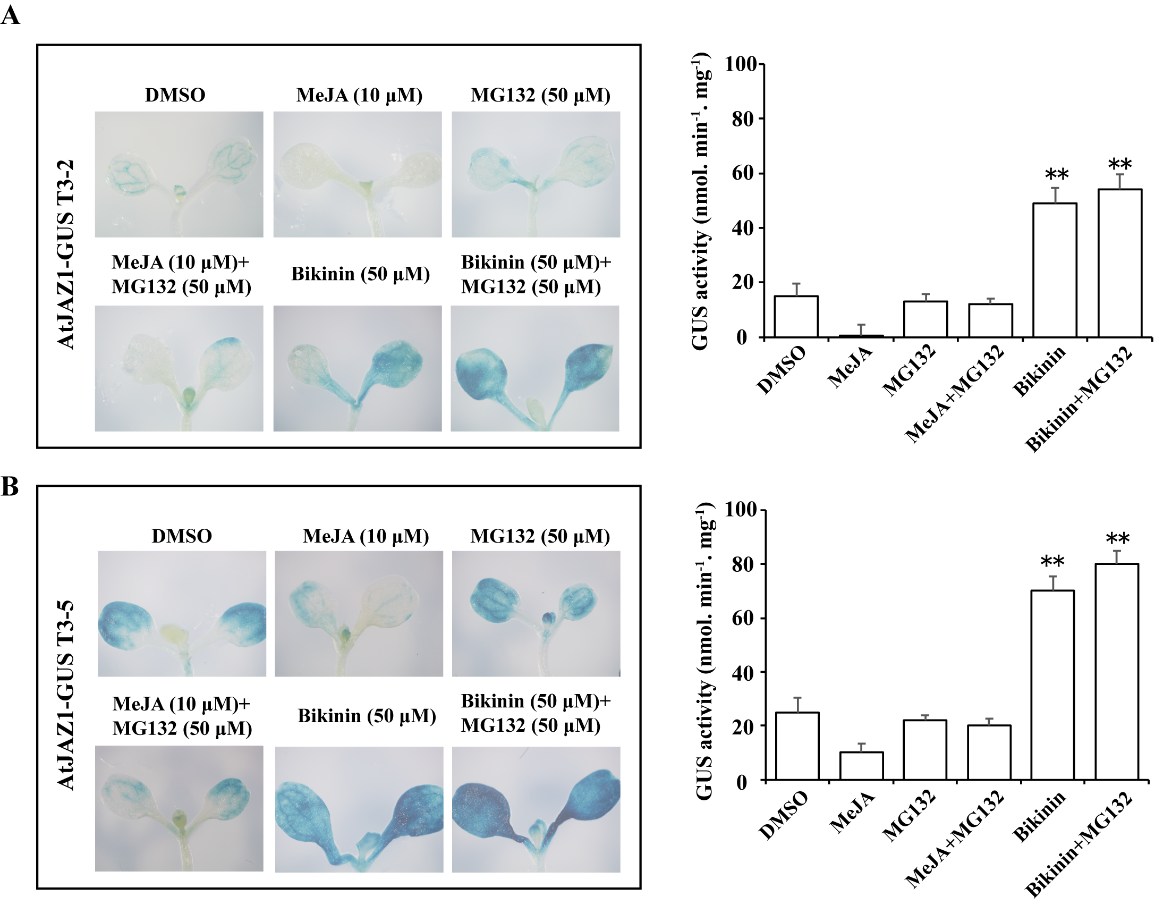


**Figure S5. BIN2 induces the degradation of AtJAZ1**

(A) *35S-AtJAZ1-GUS* T3-2 overexpression transgenic lines were treated with DMSO, MeJA (10 μM), MG132 (50 μM), MeJA (10 μM)+MG132 (50 μM), Bikinin (50 μM), or Bikinin (50 μM)+MG132 (50 μM) for 48 h. (B) *35S-AtJAZ1-GUS* T3-5 overexpression transgenic lines were treated with DMSO, MeJA (10 μM), MG132 (50 μM), MeJA (10 μM)+MG132 (50 μM), Bikinin (50 μM), or Bikinin (50 μM)+MG132 (50 μM) for 48 h. The leaves were used to perform the GUS staining assays.


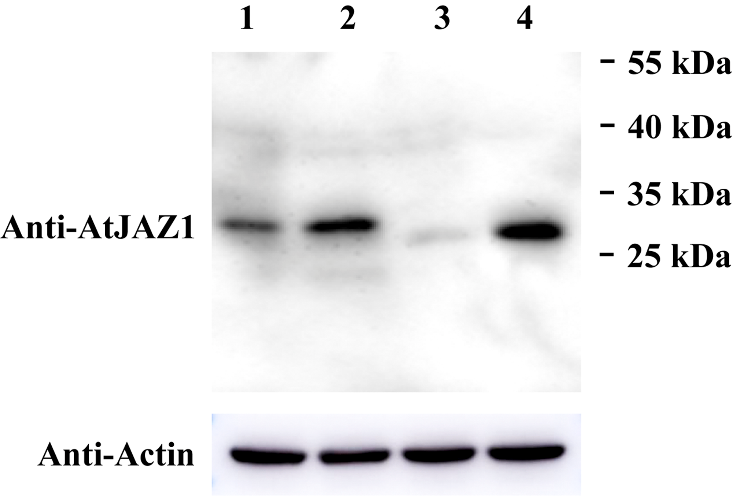


**Figure S6. Validation of AtJAZ1 antibody.**

Specificity of the AtJAZ1 antibody used in this study. Immunoblot analysis with the AtJAZ1 antibody displayed one specific band for the total protein fraction from indicated plants. The plants used are as follows: wild-type Col-0 (1), *jaz1* mutant (3), and AtJAZ1-OE lines (2 and 4). The Actin was used as a loading control.
